# Meningitic *Escherichia coli* α-hemolysin aggravates blood–brain barrier disruption *via* targeting TGFβ1-triggered hedgehog signaling

**DOI:** 10.1186/s13041-021-00826-2

**Published:** 2021-07-19

**Authors:** Jiyang Fu, Liang Li, Dong Huo, Ruicheng Yang, Bo Yang, Bojie Xu, Xiaopei Yang, Menghong Dai, Chen Tan, Huanchun Chen, Xiangru Wang

**Affiliations:** 1 State Key Laboratory of Agricultural Microbiology, College of Veterinary Medicine, Huazhong Agricultural University, Wuhan, Hubei China; 2 Key Laboratory of Preventive Veterinary Medicine in Hubei Province, The Cooperative Innovation Center for Sustainable Pig Production, Wuhan, Hubei China; 3 Key Laboratory of Development of Veterinary Diagnostic Products, Ministry of Agriculture of the People’s Republic of China, Wuhan, Hubei China; 4 International Research Center for Animal Disease, Ministry of Science and Technology of the People’s Republic of China, Wuhan, Hubei China; 5 Wuhan Animal Disease Control Center, Wuhan, Hubei China

**Keywords:** *Escherichia coli*, α-Hemolysin, Blood–brain barrier, Intercellular communication, TGFβ1, Hedgehog signaling

## Abstract

Bacterial meningitis is a life-threatening infectious disease with severe neurological sequelae and a high mortality rate, in which *Escherichia coli* is one of the primary Gram-negative etiological bacteria. Meningitic *E. coli* infection is often accompanied by an elevated blood–brain barrier (BBB) permeability. BBB is the structural and functional barrier composed of brain microvascular endothelial cells (BMECs), astrocytes, and pericytes, and we have previously shown that astrocytes-derived TGFβ1 physiologically maintained the BBB permeability by triggering a non-canonical hedgehog signaling in brain microvascular endothelial cells (BMECs). Here, we subsequently demonstrated that meningitic *E. coli* infection could subvert this intercellular communication within BBB by attenuating TGFBRII/Gli2-mediated such signaling. By high-throughput screening, we identified *E. coli* α-hemolysin as the critical determinant responsible for this attenuation through Sp1-dependent TGFBRII reduction and triggering Ca^2+^ influx and protein kinase A activation, thus leading to Gli2 suppression. Additionally, the exogenous hedgehog agonist SAG exhibited promising protection against the infection*-*caused BBB dysfunction. Our work revealed a hedgehog-targeted pathogenic mechanism during meningitic *E. coli*-caused BBB disruption and suggested that activating hedgehog signaling within BBB could be a potential protective strategy for future therapy of bacterial meningitis.

**Supplementary Information:**

The online version contains supplementary material available at 10.1186/s13041-021-00826-2.

## Introduction

Bacterial meningitis is an important life-threatening infection in the central nervous system (CNS), especially in newborn infants, young teenagers, and the elder with low immunity [1–3]. *Escherichia coli* is the most common Gram-negative bacillary organism that causes meningitis [4]. Most cases of *E. coli* meningitis initiate from the hematogenous spread and develop as circulating pathogenic bacteria penetrate and breakdown the blood–brain barrier (BBB), destroy the brain parenchyma and thus cause CNS disorders [5].

BBB is a specialized structure composed of brain microvascular endothelial cells (BMECs), astrocytes, and pericytes. This barrier separates the brain from the bloodstream and maintains the CNS homeostasis [6–8]. Among these component cells, BMECs act as the first and direct barrier unit to determine the BBB function [9, 10]. In decades, multiple effectors have been reported to participate in barrier function regulation. For instance, GDNF activated the GFRα1 and led to higher trans-endothelial electrical resistance (TEER) and lower permeability of BMECs [11]. Also, Ang1/Tie2 and Flk1 were reported to promote the capillary tube-like formation of BMECs [12]. We previously demonstrated that astrocytes-derived transforming growth factor-β1 (TGFβ1) enhanced the endothelial ZO-1 expression and maintained the BBB integrity by triggering a non-canonical hedgehog signaling in BMECs, indicating that the TGFβ1-mediated intercellular communication between astrocytes and BMECs is beneficial for BBB integrity maintaining [13], and exogenous TGFβ1 addition would exhibit a protective effect on BBB. However, whether such TGFβ1-mediated intercellular cross-talking within BBB could be hijacked during meningitic *E. coli* infection is entirely unknown.

*E. coli* α-hemolysin (HlyA), a kind of Repeats-in-toxin (RTX) exoprotein, of which synthesis, activation and secretion are regulated by the *hlyCABD* operon [14]. The HlyB and HlyD act as the transporters which belong to ATP binding cassette (ABC) superfamily and the membrane fusion protein (MFP) family, respectively. The precursor pro-HlyA is acylated by HlyC, a fatty acid acyltransferase, and transferred outside the cells by HlyB and HlyD. The posttranslational acylation of HlyA by HlyC is determinative for the cytotoxic activity [15, 16]. HlyA is largely identified in 40-50 % uropathogenic *E. coli* (UPEC) strains, such as the CFT073, J96, and UTI89 [17]. It has been demonstrated that the HlyA in UPEC was involved in inflammation activation and cell death in macrophages [18], and the HlyA was shown to induce bladder epithelial cell exfoliation and urinary tract infection [17]. Meanwhile, a variety of hemolysin toxins also played essential roles in other bacterial pathogens. In *Staphylococcus aureus*, the hemolysin induced the disseminated intravascular coagulation and liver injury [19]. In *Listeria monocytogenes*, the hemolysin LLO containing PEST-sequence co-opted the host endocytosis machinery, protecting the integrity of the host plasma membrane and enabling the growth of bacteria in host cell cytosol [20]. Unfortunately, except for UPEC, the HlyA function in meningitic *E. coli* infection was poorly investigated so far.

In this study, we demonstrated the meningitic *E. coli* interference of TGFβ1-mediated intercellular communication between astrocytes and BMECs. The α-hemolysin HlyA in meningitic *E. coli* was shown to decrease the TGFβ1 receptor TGFBRII and the key transcription factor Gli2 of hedgehog signaling, which finally led to BBB disruption. Together with our recent conclusion that astrocytes-derived TGFβ1 facilitates BBB function *via* activating non-canonical hedgehog signaling in BMECs [13], we here revealed a novel strategy for meningitic *E. coli* induction of BBB dysfunction by disturbing the regular astrocytes-endothelium cross-talking. This finding could largely extend the current knowledge of bacterial-caused CNS dysfunction from perspective of intercellular communication within BBB, and shall be beneficial for future prevention and control of bacterial meningitis.

## Methods

### Bacterial strain and cell culture

*E. coli* strain RS218 (O18:K1:H7) was originally obtained from the cerebrospinal fluid of a neonate with meningitis and gifted from Prof. Kwang Sik Kim in Johns Hopkins University School of Medicine. *E. coli* strain was grown aerobically at 37 °C in Luria-Bertani medium overnight.

The hBMECs were kindly gifted from Prof. Kwang Sik Kim in Johns Hopkins University School of Medicine, and routinely cultured in RPMI 1640 supplemented with 10 % fetal bovine serum (FBS), 2 mM L-glutamine, 1 mM sodium pyruvate, essential amino acids, nonessential amino acids, vitamins, and penicillin and streptomycin (100 U/mL). The HEK-293 T cells (ATCC® CRL-3216™) were cultured in Dulbecco’s Modified Eagle’s Medium (DMEM) with 10 % FBS and penicillin and streptomycin (100 U/mL). All cells were cultured in a 37 °C incubator under 5 % CO_2_ until reaching monolayer confluence. In some experiments, confluent hBMECs were starved in serum-free medium (1:1 mixture of Ham’s F-12 and 199 medium) for 12–16 h before further treatment.

### Reagents and antibodies

The hedgehog pathway agonist SAG and protein kinase A (PKA)inhibitor H89 were purchased from MedchemExpress (Princeton, NJ, USA). The immunofluorescence (IF) staining kits containing Cy3-labeled goat anti-rabbit IgG and FITC-labeled goat anti-rabbit IgG, 4’,6-diamidino-2-phenylindole (DAPI) reagent, EGTA, and Fluo-3-AM probe were obtained from Beyotime (Shanghai, China). Anti-Gli1, anti-Gli2, and anti-ZO-1 antibodies were from Proteintech (Chicago, IL, USA). The anti-Sp1 antibody, HRP-conjugated anti-rabbit IgG antibody, HRP-conjugated anti-mouse IgG antibody, and SimpleChIP® Plus Enzymatic Chromatin IP Kit (Magnetic Beads) were purchased from Cell Signaling Technology (Danvers, MA, USA). Anti-ZO-1 antibody for IF was from Abcam (Cambridge, MA, USA). Anti-β-actin antibody was obtained from HuaAn Biotechnology Co., Ltd. (Hangzhou, China). The lipofectamine 3000 transfection reagent was obtained from Invitrogen (Carlsbad, CA, USA). Mouse recombinant TGFβ1 was obtained from R&D system (Minneapolis, MN, USA). Evan’s blue dye was purchased from Santa Cruz Biotechnology (Santa Cruz, CA, USA).

### Mice infection assays

The 21-day-old specific-pathogen-free (SPF) female Kunming mice were obtained from the experimental animal center at China Three Gorges University (Hubei Province, China). For the infection, mice were challenged with *E. coli* strain RS218 *via* tail vein at 3 × 10^6^ CFUs. The brains from moribund and control mice were subjected to IF or Western blot assays. In some assays, the recombinant TGFβ1 protein or SAG was injected through the tail vein 12 h before or synchronously with the *E. coli* challenge as indicated.

### Western blot

Mice brains or hBMECs cultures were homogenized or lysed in RIPA buffer containing protease inhibitor cocktail and centrifuged at 15,000 g for 30 min at 4 °C to remove the insoluble cell debris. Protein concentrations of brain lysates or cell lysates were measured with a BCA protein assay kit (NCM Biotech, China), and equivalent protein samples were subjected to Western blot assay as previously described [21].

### RT-PCR and qPCR

Total RNA was extracted by the TRIzol reagent (Thermo Fisher Scientific, Waltham, MA, USA), and the RNA purity and concentration were assessed by NanoDrop 2000 Ultramicro spectrophotometer (Thermo Fisher Scientific). RT-PCR was performed to generate cDNA using HiScript II Q RT SuperMix for qPCR (+ gDNA wiper) (Vazyme, Nanjing, China). The qPCR was performed with qTOWER^3^/G quantitative real-time PCR thermal cycler (Analytikjena, Jena, Germany) using MonAmp SYBR Green qPCR Mix (Monad Biotech Co., Ltd, Wuhan, China) following the manufacturer’s instructions. The primers used for qPCR were listed in Additional file 1: Table S2. Expression of the target genes was normalized against *GAPDH*. Each assay was performed in triplicate.

### Immunofluorescence (IF)

For IF, paraffin sections of the challenged mice brains were deparaffinized and rehydrated in xylene and ethanol. IF experiments were performed according to the instructions provided by the relevant kits. Briefly, sections were washed with PBS three times and then fixed with 4 % paraformaldehyde for 30 min. The fixed cells or sections were then treated with 1 % Triton X-100 in PBS prior to non-specific site blocking and antibody incubation. Here, TGFBRII, Gli2, and ZO-1 were labeled with Cy3, and CD31 was labeled with FITC. The sections were observed with the ECHO REVOLVE microscope (Echo Laboratories, San Diego, USA).

### Electric cell-substrate impedance sensing

Electric cell-substrate impedance sensing (ECIS) Zθ system (Applied BioPhysics, NY, USA) was employed to monitor the barrier function of hBMECs with specific treatments as previously reported [22]. Briefly, cells were seeded on the collagen-coated and gold-plated electrodes in 96-well chamber slides (96W1E+) at 7 × 10^4^ cells per well and cultured until reaching confluence. The TEER was continuously monitored to reflect the formation of the barrier. After stable maximal resistance was reached, the specific reagents or treatments were added into the wells at indicated concentration, and the TEER changes were automatically monitored by the ECIS system. All data recorded in the ECIS system were analyzed and normalized as the Rb values (Norm. Parameter Values), representing the barrier function alteration along with time. Each treatment was performed with 5 parallel duplications.

### CRISPR/Cas9 genomic editing

For CRISPR/Cas9 deletion in prokaryotic cells, the α-hemolysin operon genes *hlyC*, *hlyA*, *hlyB*, and *hlyD* in RS218 were knocked out *via* CRISPR/Cas9 following the previous description [23]. In brief, the left homologous arm (HA-L) and right homologous arm (HA-R) of the *hly* genes were cloned respectively from RS218 genomic DNA and combined as the donor DNA (HA) through fusion PCR amplification. The corresponding sgRNA was synthesized and inserted into plasmid pTargetF (Addgene: #62,226) *via* inverse-PCR. RS218 strain containing pCas plasmid (Addgene: #62,225) was then transformed with donor DNA and the pTargetF plasmid containing sgRNA sequence. The transformant was grown and screened by kanamycin (200 µg/mL) and spectinomycin (100 µg/mL), and the possible mutations were PCR identified with sequencing. Except for the deletion, the CDS region of *hlyCA* was cloned in pMD19-T Vector and transformed in the corresponding deletion mutant to complement the *hlyA* knock-out. The primers used for these genes editing were listed in Additional file 1: Table S6.

### Transfection

HEK-293 T or hBMECs cells grown to 70 % confluence were subjected to transfection experiments with Lipofectamine 3000 reagent according to the manufacturer’s instructions (Invitrogen, MA, USA). Briefly, 5 µg of plasmids, 10 µL of P3000, 7.5 µL of Lipo3000, and 500 µL of Opti-MEM were mixed gently and incubated at room temperature for 15 min. The mixture was then added dropwise to the cells in the 6-well plates and incubated at 37 ℃ with 5 % CO_2_ for 24 h. For Sp1 overexpression in hBMECs, fresh medium with G418 (1 mg/ml) was applied for another 21 days to screen and maintain the positively transfected cells.

### Dual-luciferase reporter assay

Prior to luciferase reporter assay, the coding sequence (CDS) of human Sp1 were amplified and cloned into pcDNA3.1(+) vector to generate the overexpression plasmid pcDNA3.1-Sp1. The promotor region of *tgfbr2* was amplified and cloned into the firefly luciferase reporter vector pGL3-basic to generate the wild-type reporter plasmids pGL3-tgfbr2-promo-WT. Meanwhile, a serial of truncated promoter, as well as site-directed mutation of promotor, were similarly constructed into pGL3-basic (Fig. 3). The potential binding sites were identified with the JASPAR database online (http://jaspar.genereg.net/). All primers used in the dual-luciferase assays were listed in Additional file 1: Table S3.

For dual-luciferase reporter assay, the pcDNA3.1 overexpression plasmid, the corresponding pGL3 reporter plasmid, and pRL-TK plasmid were co-transfected into HEK-293 T cells in 24-wells plates. Both firefly luciferase activity and renilla luciferase activity were tested after 36 h of transfection by Dual-Luciferase Reporter assay system (Promega, WI, USA) with Spark 10 M multimode microplate reader (Tecan, Männedorf, Switzerland). Relative luciferase activity was calculated by the ratio of reporter activity (firefly fluorescence) to that of control activity (renilla fluorescence), and the results were shown as the representative of three independent assays.

### Chromatin immunoprecipitation

Chromatin immunoprecipitation (ChIP) was performed to test the interaction between transcription factors and its potential target genes using SimpleChIP® Plus Enzymatic Chromatin IP Kit (CST) following the manufacturer’s instructions. Briefly, cells in the dishes were fixed in formaldehyde to cross-link proteins with DNAs. Cells were next digested by micrococcal nuclease and subjected to the immunoprecipitation procedure. The products were treated with protease K and then subjected to DNA isolation. Purified DNA was used in the following qPCR amplification. The primers used for ChIP-qPCR were listed in Additional file 1: Table S4.

### Bacterial infection of hBMECs

*Escherichia coli* strain RS218 infection of hBMECs was performed following our previously described methods [24]. Briefly, the confluent hBMECs were starved in serum-free medium for 12–16 h. Overnight *E. coli* cultures were resuspended and diluted in the same serum-free medium and added to the cells at a multiplicity of infection of 100 for the indicated time points. Cells were then washed three times with pre-chilled PBS and collected for RNA isolation using TRIzol reagent or protein extraction with RIPA lysis buffer.

### Construction and screening of RS218 Tn5-transposon mutant library

The Tn5-transposon mutant library of RS218 strain was constructed with pUTmini-Tn5 Cm Kit following the protocol (Biomedal, Spain). The pUTmini-Tn5 Cm plasmid was transformed into RS218 through conjugation transfer with the donor strain *E. coli* X7213. The transformants were screened on plates containing chloramphenicol at 50 µg/mL to select the positive insertions, as we previously reported [25]. Next, the mutants in this library were subjected to the firefly luciferase activity screening, specifically targeting *gli2* promotor activity. Briefly, each RS218 mutant was inoculated into HEK-293 T cells containing pGL3-*gli2*-promo-WT in 96-wells plates and incubated for 2 h to establish the infection, with the wild-type RS218 (RS218-WT) as the positive control. The luciferase activities in all wells were high-throughput measured with the Spark 10 M multimode microplate reader by using Luc-Pair™ Firefly Luciferase HS Assay Kit (iGene Biotechnology Co., Ltd., China) following the instruction. The mutants that could not significantly attenuate the luciferase activity were picked and double-checked, and the genomic DNA was extracted to amplify and analyze the flanking sequence of Tn insertion sites through the thermal asymmetric interlaced PCR (TAIL-PCR) as reported previously [26, 27]. Primers used for TAIL-PCR were listed in Additional file 1: Table S5.

### Expression and purification of the recombinant HlyA

HlyA (the active α-hemolysin) and pro-HlyA (inactive α-hemolysin precursor) were cloned and expressed as previously reported [28]. Briefly, the CDS of the *hlyA* gene and the CDS of *hlyCA* were cloned into the pET-28a(+) vector to generate the pET28a-*hlyA* and pET28a-*hlyCA* expression plasmids. An additional T7 promotor-lacO element was introduced between *hlyC* and *hlyA* in pET28a-*hlyCA*, so that *hlyC* and *hlyA* genes were under the same control of transcription and translation initiation signals. The HlyA and pro-HlyA proteins were expressed in *E. coli* BL21(DE3) strain under the induction of 1 mM isopropyl β-D-1-thiogalactopyranoside (IPTG) for 4 h at 37 ℃. The products were finally solubilized and purified with the Ni-NTA agarose column. Primers used for the cloning were listed in Additional file 1: Table S7.

### Intracellular Ca^2+^ determination

The confluent hBMECs were starved in serum-free medium for 16 h and then in medium containing Fluo-3-AM (5 µM) for another 1 h. The medium was replaced by the complete medium and incubated at 37 ℃ for 20 min. Subsequently, the cells were challenged with RS218 or the recombinant HlyA/pro-HlyA protein at the indicated dose for 2 h. The cells were observed with the ECHO REVOLVE microscope, and the fluorescence intensity of intracellular Ca^2+^ level was measured with Spark 10 M multimode microplate reader or analyzed through BD FACSVerse™ flowcytometry (BD, CA, USA).

### PKA activity test

PKA activity was measured using the PepTag Non-Radioactive Protein Kinase Assay specific for PKA (Promega, WI, USA) following the manufacturer’s instructions. Briefly, cells were challenged with RS218 or treated with recombinant HlyA/pro-HlyA protein, and then collected and homogenized in PKA extraction buffer. The lysates were centrifuged, and the supernatant was incubated with the reaction mix for another 30 min. Samples were finally separated on the 0.8 % agarose gel for 20 min to analyze the activity reflected by the P-peptide.

#### In vivo BBB permeability assay

BBB permeability was evaluated using Evan’s blue dye (961 Da) as we previously described [29]. Briefly, mice were intravenously challenged by bacterial strains for 6 h or the indicated SAG for 12 h. After that, 500 µL Evan’s blue (5 mg/mL) was injected *via* the tail vein to allow circulation for 10 min before mice being sacrificed and perfused. Brains were collected and photographed for extravascular staining of the dye.

### Statistical analysis

Data were expressed as the mean ± standard error of the mean (mean ± SEM) from at least three replicates. Statistical significance of each group’s differences was analyzed by a one-way analysis of variance (ANOVA) or two-way ANOVA embedded in GraphPad Prism, version 6.0 (GraphPad Software Inc., La Jolla, CA, USA). *P* < 0.05 (*) was considered significant, and *p* < 0.01 (**) was considered extremely significant.

## Results

### Meningitic ***E. coli*** disturbed the astrocytes-endothelium communication by attenuating TGFβ1-mediated non-canonical hedgehog signaling

**Fig. 1:**
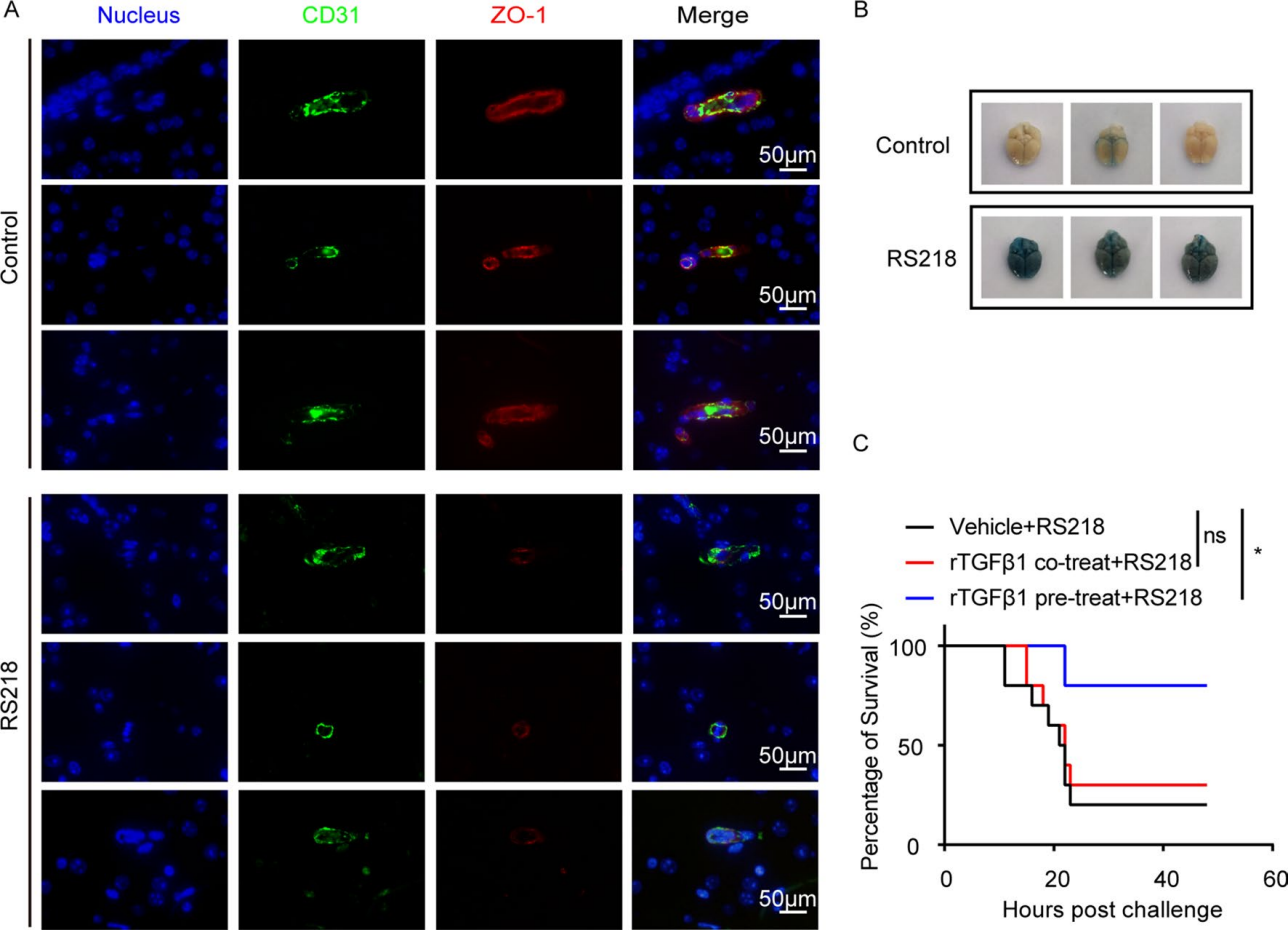
Meningitic *E. coli* infection broke the BBB integrity and caused death in challenged mice. **A** The expression of ZO-1 on BMECs of the mice in response to RS218 infection *via* IF. The BMECs were marked with CD31 in green. Scale bars indicated 50 μm. **B** Evan’s blue assay assessing the BBB permeability upon RS218 infection (n = 3). **C** Effects of the rTGFβ1 pre-treatment (for 12 h) or co-treatment (synchronously) (at 1 µg/kg) on the survival of mice challenged with RS218 (n = 10). Survival data were collected and shown as Kaplan–Meier survival curves, and the statistical analysis was carried out by Log-rank (Mantel–Cox) test. *p < 0.05

We have previously demonstrated that meningitic *E. coli* infection could break the tight junctions and caused BBB integrity disruption [21, 29]. Here, we further validated this phenotype by challenging the mice with meningitic *E. coli* strain RS218. The IF assay showed that the ZO-1 in BMECs of the infected mice was largely decreased compared to the control mice (Fig. 1A), and the BBB permeability was obviously increased in the challenged mice brain, evaluated by Evan’s blue dye infiltration (Fig. 1B). Noticeably, we have recently evidenced that astrocytes-derived TGFβ1 could facilitate the BBB barrier function by increasing ZO-1 expression in BMECs *via* a non-canonical hedgehog signaling [13]. We therefore presumed that such TGFβ1-mediated intercellular communication between astrocytes and endothelium was largely disturbed during meningitic *E. coli* infection. To verify this hypothesis, we treated mice with rTGFβ1 (i.v.) prior to or along with meningitic *E. coli* RS218 injection. As presented in Fig. 1C, the mice pretreated with rTGFβ1 prior to infection were largely protected from death (8 of 10 were survived), while most mice receiving rTGFβ1 synchronously at the infection failed to survive (3 of 10 were survived), exhibiting a similar survival rate as the challenged mice without any treatment (2 of 10 were survived). These outcomes reflected that strengthen the BBB integrity by rTGFβ1 prior to bacterial challenge could well protect mice from death, while in contrast rTGFβ1 co-treating with the infection could not provide mice the effective protection. Meningitic *E. coli* could subvert this TGFβ1-regulated barrier homeostasis during the infection.

**Fig. 2:**
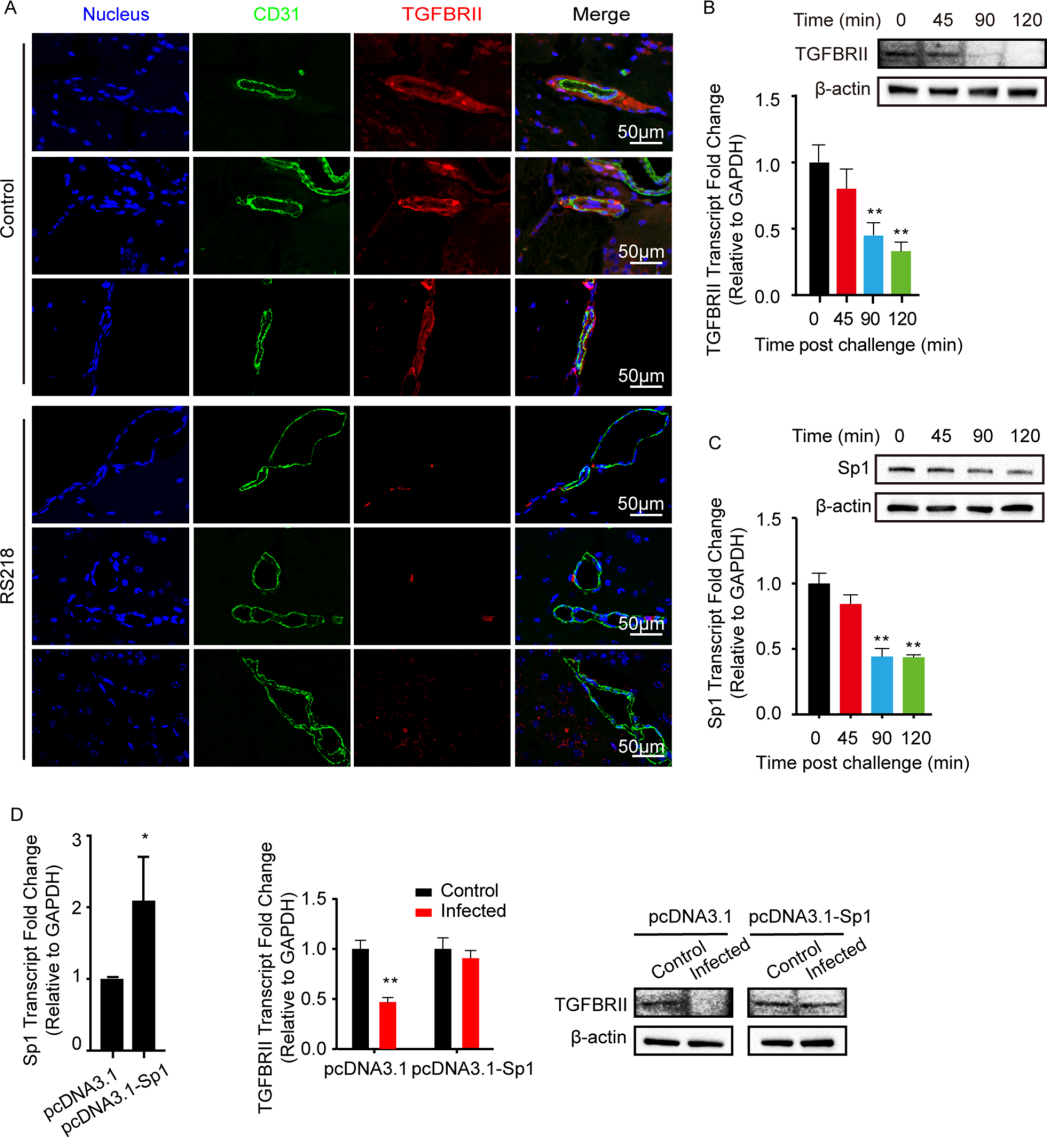
Meningitic *E. coli* infection specifically suppressed TGFBRII expression in BMECs *via* attenuated Sp1. **A** The expression of TGFBRII on BMECs of the mice in response to RS218 infection *via* IF. The BMECs were marked with CD31 in green. Scale bars indicated 50 μm. **B**, **C** Expression alteration of TGFBRII (**B**) or Sp1 (**C**) in hBMECs along with RS218 infection at indicated time points. **p < 0.01. **D** The effect of Sp1 overexpression on the RS218 infection-caused TGFBRII reduction in hBMECs by qPCR and Western blot. The Sp1 was cloned in pcDNA3.1(+) and transfected into hBMECs for the overexpression (left panel). **p < 0.01. The qPCR assays were performed in triplicates, and results were presented as mean ± SEM

To address this concern, we firstly analyzed the expression of the TGFβ1 receptor on BMECs of the mice challenged with meningitic *E. coli* RS218 *via* IF. As shown, meningitic *E. coli* infection significantly decreased the expression of TGFBRII on the BMECs (labeled with CD31) of the RS218-challenged mice (Fig. 2A). *In vitro*, RS218 infection also caused a significant decrease of TGFBRII in hBMECs (Fig. 2B). We next predicted the Sp1 that would act as the potential transcription factor targeting TGFBRII promotor, and both Western blot and qPCR data showed that Sp1 was also decreased in hBMECs along with RS218 infection (Fig. 2C). By overexpression of Sp1 in hBMECs (Fig. 2D, left panel), we further observed that the infection-caused TGFBRII reduction was completely restored detected by Western blot and qPCR (Fig. 2D). These observations indicated that transcription factor Sp1 was involved in meningitic *E. coli*-induced TGFBRII decrease.

**Fig. 3:**
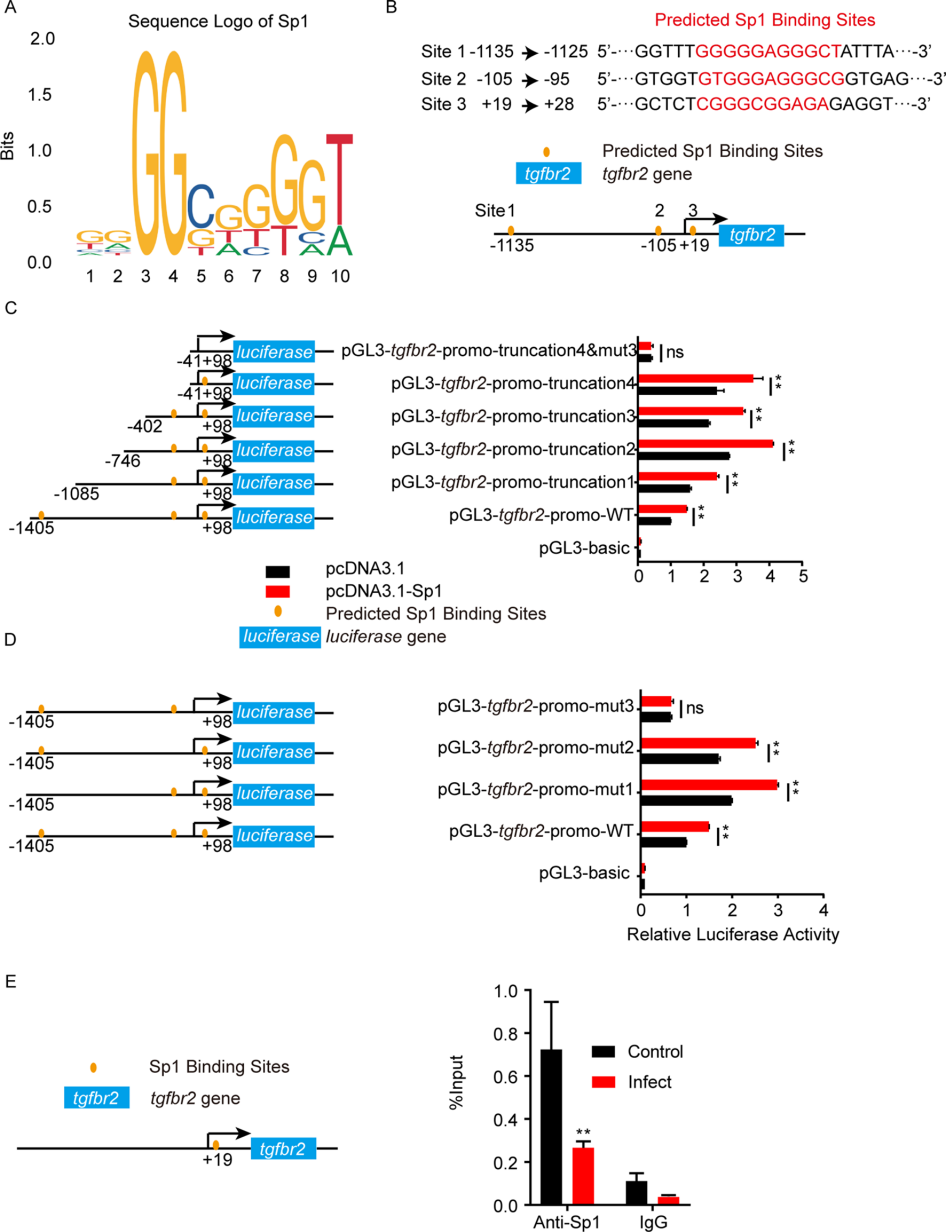
Dual-luciferase reporter assays detected Sp1 binding sites on *tgfbr2* promotor. **A** The sequence logo of the Sp1 binding motif. **B** Schematic of the 3 predicted Sp1 binding sites on *tgfbr2* promotor and their binding sequences accordingly. The binding sites were located at -1135 to -1125 (site 1), -105 to -95 (site 2) and + 19 to + 28 (site 3) of the *tgfbr2* promotor. The *tgfbr2* luciferase activities tested by applying a series of truncations (**C**) as well as site-targeted mutations (**D**) on the *tgfbr2* promoter, along with pcDNA3.1-Sp1 and pRL-TK plasmids. The specific constructs used in the truncation assays (**C**) included pGL3-basic vector, pGL3-*tgfbr2*-promo-WT (containing promotor region from − 1405 to + 98), pGL3-*tgfbr2*-promo-truncation1 (from − 1085 to + 98), pGL3-*tgfbr2*-promo-truncation2 (from − 746 to + 98), pGL3-*tgfbr2*-promo-truncation3 (from − 402 to + 98), pGL3-*tgfbr2*-promo-truncation4 (from − 41 to + 98) and pGL3-*tgfbr2*-promo-truncation4&mut3 (from − 41 to + 98 which lacking site 3). The specific constructs used in the site-mutation assays (**D**) included pGL3-basic vector, pGL3-*tgfbr2*-promo-WT (containing all 3 sites), pGL3-*tgfbr2*-promo-mut1 (lack of site 1), pGL3-*tgfbr2*-promo-mut2 (lack of site 2), or pGL3-*tgfbr2*-promo-mut3 (lack of site 3). The luciferase activities were determined and presented as the ratio of firefly luciferase activity and renilla luciferase activity. The assays were performed with 3 replicates, and data were presented as mean ± SEM. **p < 0.01. ns, no significance. **E** Schematic of the evidenced Sp1 binding site on *tgfbr2* promotor and ChIP-qPCR verification of Sp1 binding with the *tgfbr2* promotor at the site around + 19 in hBMECs upon infection. The ChIP procedure was performed with an anti-Sp1 antibody, and rabbit IgG was employed as the negative control. The qPCR was assayed with 3 replicates, and data were presented as mean ± SEM. **p < 0.01

According to Sp1 binding motif (Fig. 3A), there were 3 potential Sp1 binding sites on the *tgfbr2* promotor region (Fig. 3B). Since functioning as the transcription factor, we next validated these potential Sp1 binding sites on the *tgfbr2* promotor by the dual-luciferase reporter assays. The 3 predicted Sp1 binding sites on *tgfbr2* promotor region (site 1–3) were shown in Fig. 3B. The *tgfbr2* promotor regions, including the full-length promotor region and a series of truncations and site-mutations, were cloned and constructed. Dual-luciferase reporter assays from both truncations and site-mutations clearly indicated that the site 3, 5’-CGGGCGGAGA-3’ (from + 19 to + 28), was the Sp1 binding region on *tgfbr2* promotor (Fig. 3C and D). Besides, through ChIP-qPCR with anti-Sp1 antibody, the flanking sequences of the site 3 were positively detected, and Sp1 binding with this promotor region in hBMECs was also significantly decreased upon RS218 infection (Fig. 3E). These results together suggested that meningitic *E. coli* infection decreased the TGFBRII expression in hBMECs through Sp1-regulated *tgfbr2* promotor, thus disturbing the TGFβ1-mediated astrocytes-endothelium communication.

**Fig. 4:**
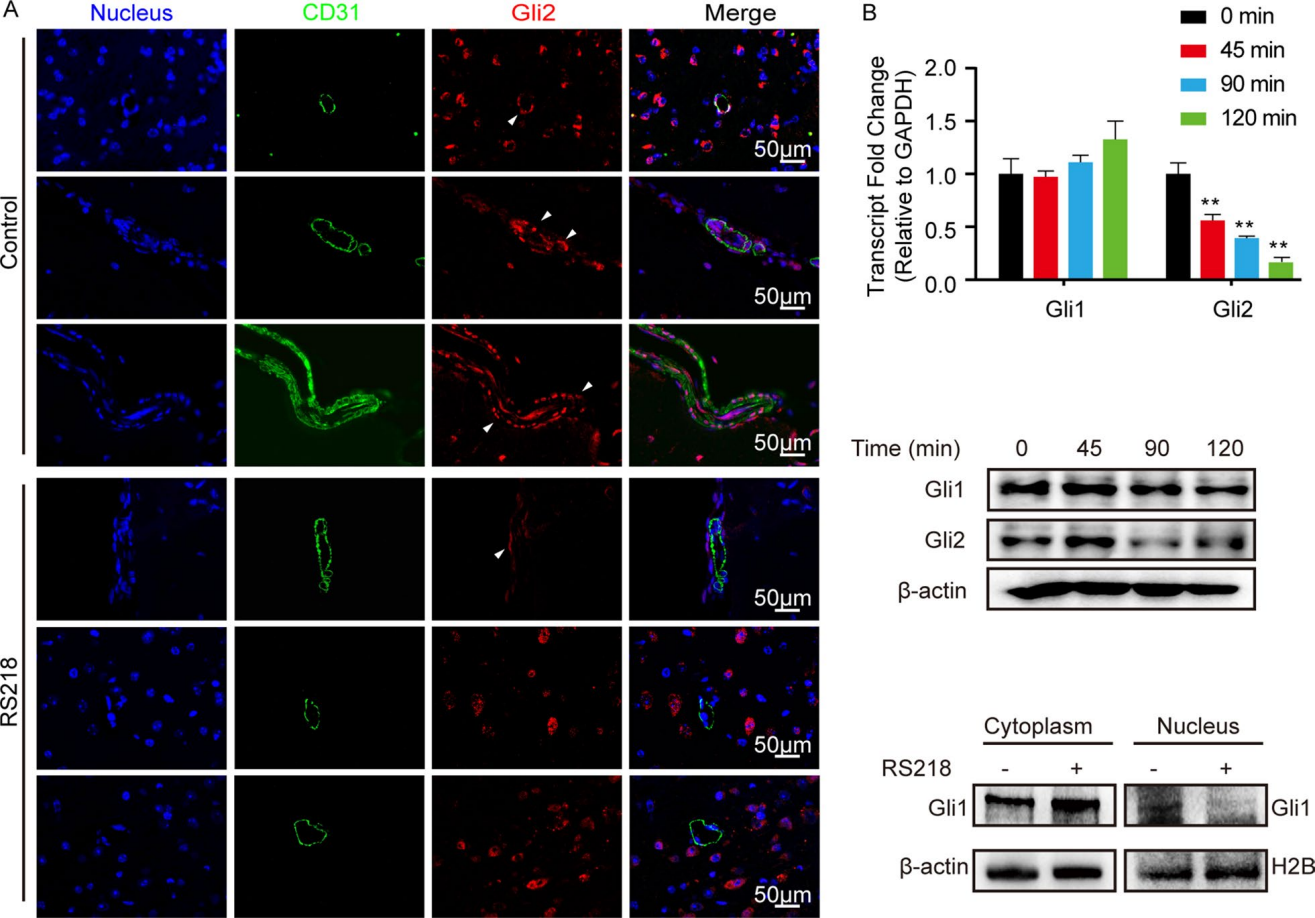
Meningitic *E. coli* infection attenuated the hedgehog signaling in BMECs. **A** The expression of Gli2 on BMECs of the challenged mice *via* IF. The BMECs were marked with CD31 in green. White triangles indicated Gli2 in BMECs. Scale bars indicated 50 μm. **B** Expression alterations of Gli1 and Gli2 in hBMECs along with RS218 infection *via* qPCR and Western blot, and the subcellular localization of Gli1 in hBMECs 2 h post infection. **p < 0.01. The qPCR assays were performed in triplicates, and results were presented as mean ± SEM

Previously we have evidenced that astrocytes-derived TGFβ1 facilitated the BBB barrier function in BMECs *via* a non-canonical hedgehog signaling [13]. Here, our *in vivo* assay also demonstrated that the hedgehog signaling transcription factor Gli2 in mice BMECs was largely reduced in response to RS218 infection (Fig. 4 A). And the *in vitro* qPCR and Western blot assay also showed a time-dependent reduction of Gli2 in hBMECs upon the infection (Fig. 4B). Although we here did not observe the significant decrease of Gli1 expression upon the infection, the nucleus/cytoplasm extraction clearly showed that the nucleus-located Gli1 significantly decreased in response to RS218 infection (Fig. 4B). These findings indicated that the hedgehog signaling transcription factor Gli1/2 was hijacked by meningitic *E. coli* for disturbing this astrocytes-endothelium communication. However, how meningitic *E. coli* targets Gli1/2 is yet to be investigated.

### α-Hemolysin was the primary virulence determinant responsible for RS218-caused Gli2 and ZO-1 decrease in hBMECs

**Fig. 5:**
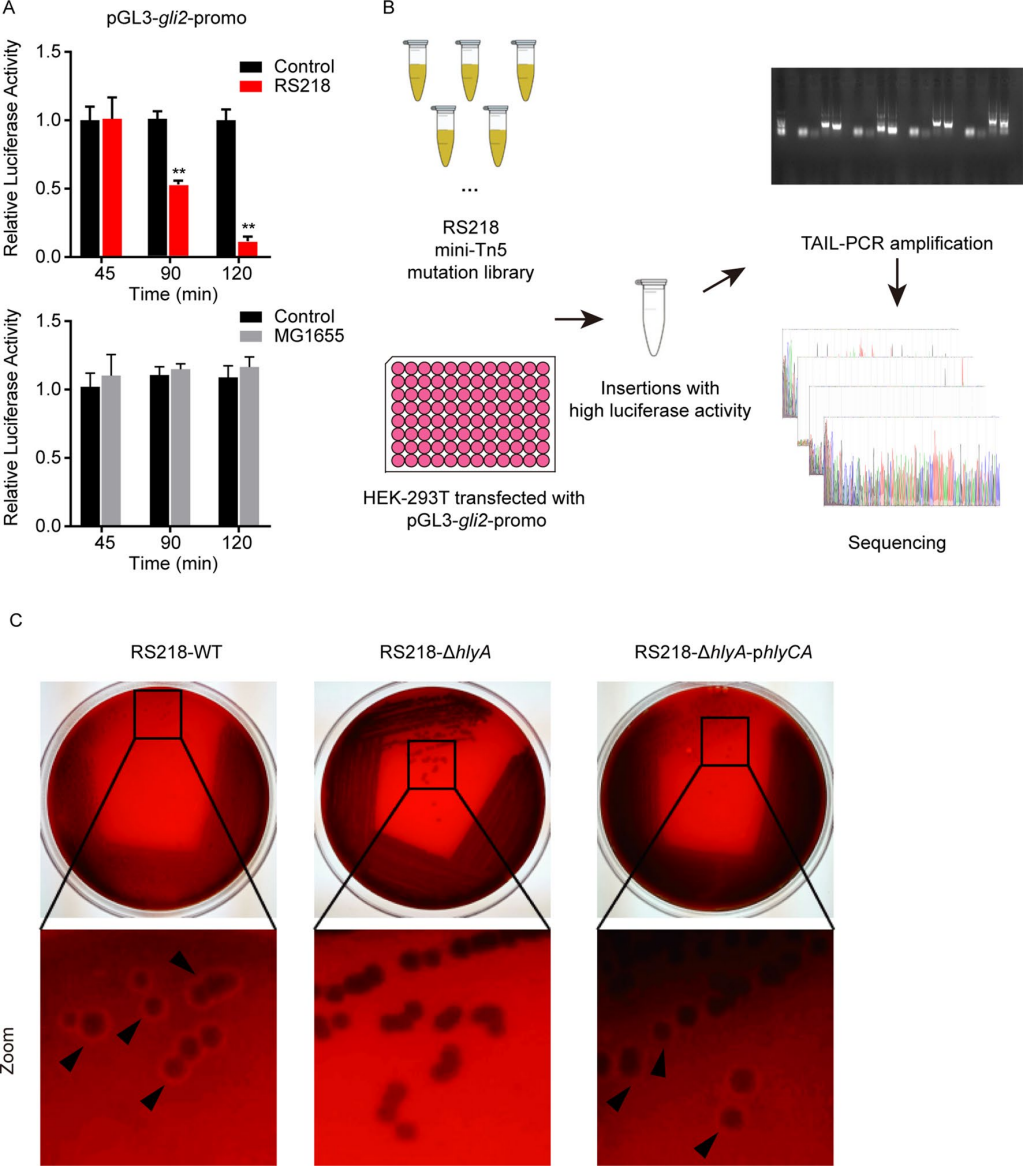
High-throughput screening of Tn5-transposon mutation library identified α-hemolysin that regulating Gli2 transcription in hBMECs. **A** Firefly-luciferase reporter assay testing the effect of RS218 infection on the *gli2* promoter activity. *E. coli* K12 strain MG1655 was compared as the control. Results were obtained from three independent assays and presented as mean ± SEM. **p < 0.01. **B** Schematic diagram showing the screening process of Tn5-transposon mutation library in RS218. **C** Hemolysis rings of RS218-WT, RS218-Δ*hlyA*, and RS218-Δ*hlyA*-p*hlyCA* growing on sheep blood agar plates

Subsequently, we attempted to explore the mechanism of meningitic *E. coli* targeting Gli1/2. Since Gli2 as well as the gene promotor activity in hBMECs was observed to be significantly decreased by meningitis *E. coli* RS218 (Fig. 4B and Additional file 1: Fig S1A), we therefore transfected the pGL3-gli2-promo-WT reporter plasmid into HEK-293 T cells, and preliminarily tested the regulative effect of RS218 on Gli2 transcription. As shown, the *gli2* luciferase activity exhibited a significantly time-dependent decrease in response to meningitic *E. coli* RS218 infection. In contrast, the non-meningitic *E. coli* K12 strain MG1655 did not show any decreased effect on the *gli2* luciferase activity (Fig. 5 A). This data indicated the availability of this screening approach and suggested that there should be some specific virulence determinants responsible for the decrease of Gli2. Next, our previously generated Tn5 transposon mutation library in RS218 was applied for high-throughput screening of the bacterial determinants associated with Gli2 activity. The mutations inducing higher luciferase activity compared with the RS218-WT were picked. The sequences flanking the transposon insertion site were amplified with TAIL-PCR, and the products were subjected to sequencing and genome alignment (Fig. 5B). A total of 40 Tn5-mutations were found unable to decrease but induced much higher *gli2* promotor luciferase activity than the RS218-WT (Additional file 1: Fig. S1B). After sequencing and duplicates removal, 29 distinct mutations were finally identified (Table S1), and 23 of them were identified as α-hemolysin operon *hlyCABD* insertions (Additional file 1: Fig. S1C). Among these 23 mutations, the CDS regions of *hlyA*, *hlyB*, and *hlyD* were inserted by Tn5 transposon for 21 times, and the reported regulative region upstream of *hlyC* was inserted by Tn5 transposon twice. These results implied that α-hemolysin was likely to be an important determinant which was responsible for the downregulation of Gli2.

**Fig. 6:**
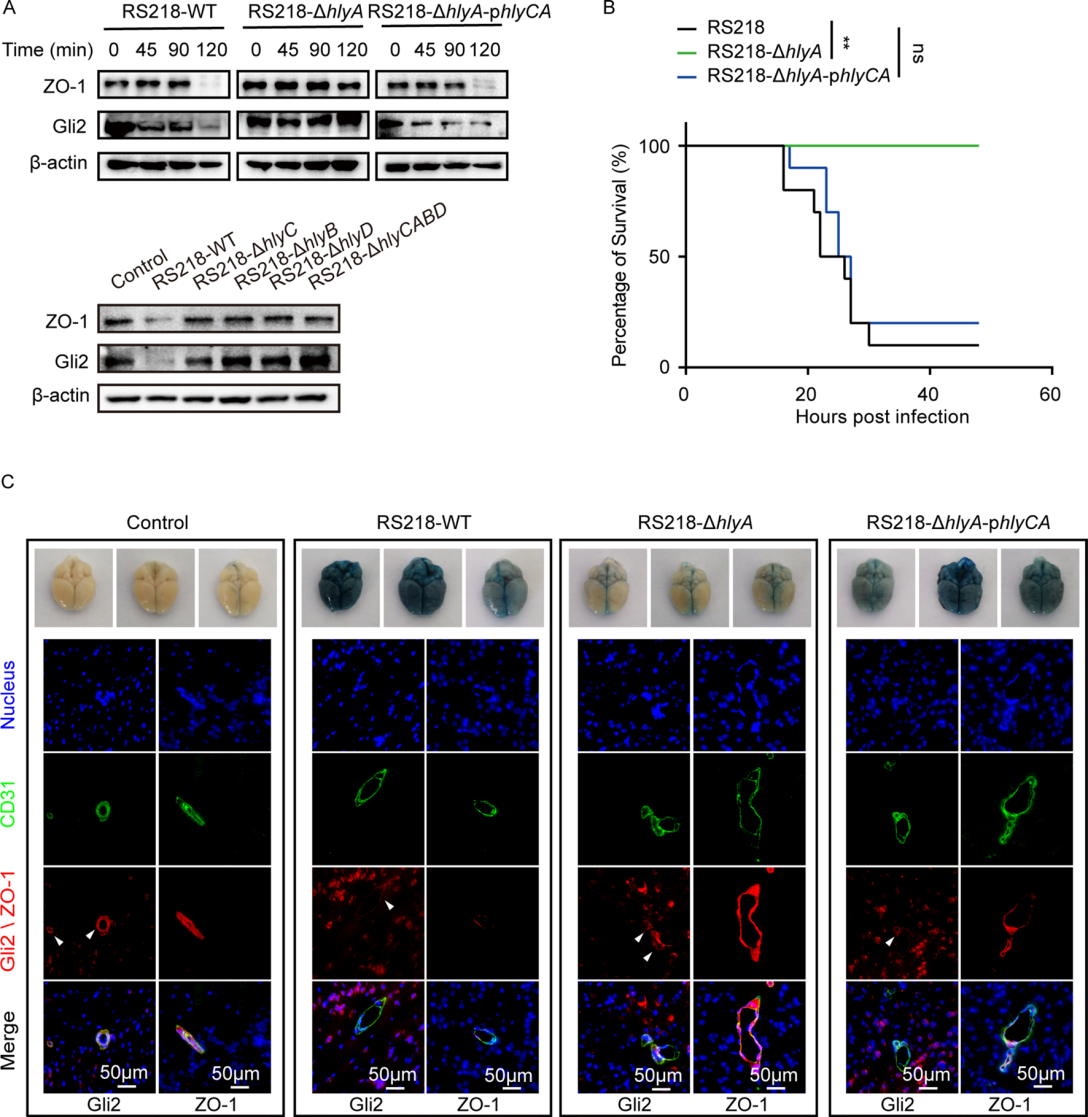
*E. coli* α-hemolysin was responsible for the infection-caused decrease of Gli2 and ZO-1 as well as the BBB integrity damage. **A** The expression of ZO-1 and Gli2 in hBMECs in response to RS218-WT, RS218-Δ*hlyA*, and RS218-Δ*hlyA*-p*hlyCA* strains, as well as other α-hemolysin operon genes mutants. **B** Mice survival upon the infection of RS218-WT, RS218-Δ*hlyA*, and RS218-Δ*hlyA*-p*hlyCA* strains (n = 10). Survival data were collected and shown as Kaplan–Meier survival curves, and the statistical analysis was carried out by Log-rank (Mantel–Cox) test. **p < 0.01. ns, no significance. **C** Evan’s blue and IF assays showing the BBB permeability and both Gli2 and ZO-1 expression in brains of mice challenged by RS218-WT, RS218-Δ*hlyA*, and RS218-Δ*hlyA*-p*hlyCA* strains. The white triangles indicated Gli2 in BMECs. Scale bar indicated 50 μm

To confirm this hypothesis, we generated a serial of deletion mutants of *hlyCABD* operon *via* CRISPR/Cas9 system and evaluated their hemolytic abilities *in vitro*. As presented in Fig. 5C, the *hlyA*-deleted strain (RS218-Δ*hlyA*, shown as the representative) obviously loss the hemolytic ability on the sheep blood agar plate compared with the wild-type strain, while the complemented strain (RS218-Δ*hlyA*-p*hlyCA*) completely restored this hemolysis phenotype (black triangles indicated the hemolysis ring), suggesting the successful genetic and functional deletion as well as complementation of *hlyA*. Moreover, we found the significantly decreased expression of ZO-1 and Gli2 in hBMECs in response to RS218-WT strain and the complement strain RS218-Δ*hlyA*-p*hlyCA*, while the mutant RS218-Δ*hlyA* did not decrease both expression (Fig. 6A). Similarly, the other α-hemolysin deletion mutants, including RS218-Δ*hlyC*, RS218-Δ*hlyB*, RS218-Δ*hlyD*, and RS218-Δ*hlyCABD* were also tested and they were all unable to decrease the expression of Gli2 and ZO-1 in hBMECs (Fig. 6A). *In vivo*, the mice challenged with RS218-Δ*hlyA* strain showed 100 % survival, while those challenged with RS218-WT and RS218-Δ*hlyA*-p*hlyCA* exhibited around 80 % death within 48 h post infection (Fig. 6B). Meanwhile, the mice BBB permeability was assessed *via* Evan’s blue assay and showed that both RS218-WT and RS218-Δ*hlyA*-p*hlyCA* strains caused the significantly increased permeability, demonstrated by the heavy Evan’s blue dye effusion out of the brain vessels. While in contrast, the mutant strain RS218-Δ*hlyA* did not show too much damage to the vessel permeability (Fig. 6C). The IF results also supported that both Gli2 and ZO-1 expression (labeled with Cy3) around the BMECs (labeled with anti-CD31-FITC) were significantly reduced by the RS218-WT and RS218-Δ*hlyA*-p*hlyCA* infection, while the RS218-Δ*hlyA* strain did not influence the expression of Gli2 and ZO-1 (Fig. 6C). Together, these findings largely evidenced that RS218 α-hemolysin was the major determinant contributing to the infection-caused Gli2 and ZO-1 decrease in BMECs.

### α-Hemolysin-triggered Ca^2+^ influx and PKA activation accounted for the decreased expression of Gli2 and ZO-1 in hBMECs

**Fig. 7:**
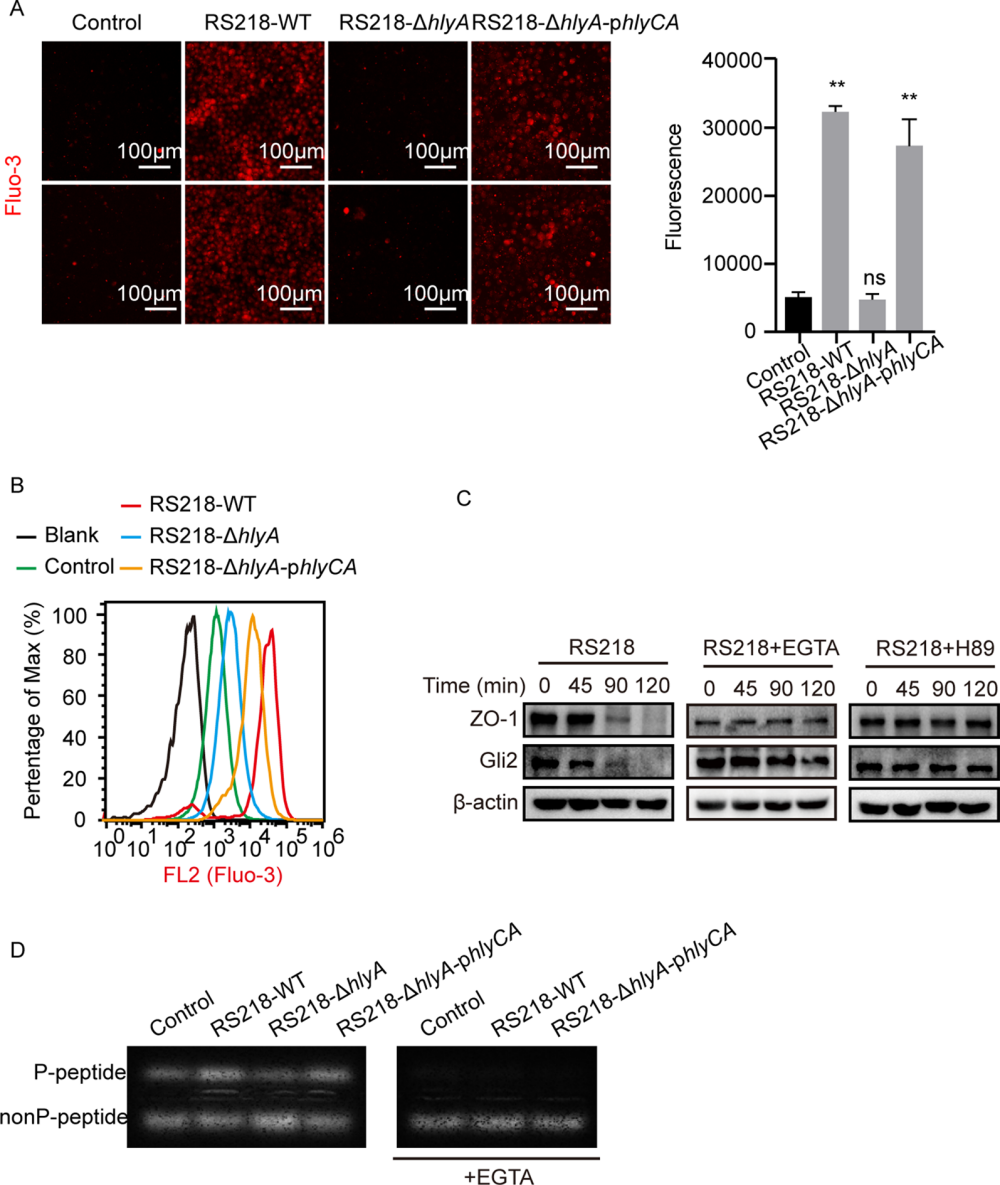
The HlyA-triggered Ca^2+^ influx and PKA activation accounted for the infection-caused ZO-1 and Gli2 reduction. **A**, **B** Ca^2+^ influx of hBMECs upon infection of RS218-WT, RS218-Δ*hlyA*, and RS218-Δ*hlyA*-p*hlyA* strains for 2 h determined by the Fluo-3-AM probed through the fluorescence microscopy (**A**) and flow cytometry (**B**). The assay was performed with 3 replicates, and data were presented as mean ± SEM. ** *p* < 0.01. ns, no significance. Scale bar indicated 100 μm. Cells in Blank group were not treated. Cells in Control group, RS218-WT group, RS218-Δ*hlyA* group, and RS218-Δ*hlyA*-p*hlyA* group were loaded with Fluo-3-AM and treated as indicated. **C** The expression of ZO-1 and Gli2 in hBMECs upon RS218 infection, as well as upon infection with pre-treatment of Ca^2+^ chelator EGTA (5 mM) or PKA activity inhibitor H89 (20 µM). **D** PKA activity in hBMECs upon the infection of RS218-WT, RS218-Δ*hlyA*, and RS218-Δ*hlyA*-p*hlyA* strains, and upon the infection in the presence of EGTA (5 mM)

The α-hemolysin HlyA was a kind of prototype RTX toxin and contained repeating RTX domains that consist of several glycine- and aspartate- rich nonapeptide units for Ca^2+^ binding [30]. We therefore presumed that Ca^2+^ played a certain role in the α-hemolysin-induced effects. By applying Fluo-3-AM, a specific probe to indicate Ca^2+^, we measured the intracellular Ca^2+^ level in hBMECs by the infection of RS218-WT, RS218-Δ*hlyA*, and RS218-Δ*hlyA*-p*hlyCA*. As shown, both RS218-WT and RS218-Δ*hlyA*-p*hlyCA* strains induced strong Ca^2+^ influx in hBMECs, while the RS218-Δ*hlyA* mutant could not increase the intracellular Ca^2+^ level (Fig. 7A). The flow cytometry also supported this observation that both RS218-WT and RS218-Δ*hlyA*-p*hlyCA* strains induced much higher levels of intracellular Ca^2+^, compared with that induced by the mutant RS218-Δ*hlyA* (Fig. 7B). In addition, when extracellular Ca^2+^ was chelated by EGTA, the RS218-induced time-dependent downregulation of ZO-1 and Gli2 was suppressed entirely (Fig. 7C). Meanwhile, we similarly tested the Ca^2+^ influx in hBMECs by the treatment of recombinant HlyA protein, including the active HlyA as well as its inactive form pro-HlyA, and results showed that the active HlyA caused a significantly increased and much higher level of intracellular Ca^2+^, compared with the treatment by pro-HlyA (Additional file 1: Fig. S2A and S2B). Consistently, the active HlyA, not the pro-HlyA, was shown to downregulate ZO-1 and Gli2 expression in hBMECs, while this HlyA-induced downregulation of ZO-1 and Gli2 were completely prevented by treating with Ca^2+^ chelator EGTA (Fig S2C).

It was known that PKA was an essential transducer of intracellular electrical activity [31] and was recognized as the suppressor of Gli2 in cells [32]. We next measured the PKA activity in hBMECs by the infection of RS218-WT, RS218-Δ*hlyA*, and RS218-Δ*hlyA*-p*hlyCA* strains. The results suggested that RS218-WT and RS218-Δ*hlyA*-p*hlyCA* infection of hBMECs largely enhanced the PKA activity, demonstrated by an increased level of P-peptide, and this increased PKA activity by both RS218-WT and RS218-Δ*hlyA*-p*hlyCA* infection could be entirely suppressed by the treatment of EGTA (Fig. 7D). However, the RS218-Δ*hlyA* strain was shown unable to effectively activate PKA activity, demonstrated by a relatively higher level of the nonP-peptide (Fig. 7D). The recombinant active HlyA was also shown to increase PKA activity by a relatively higher P-peptide level, while the pro-HlyA did not (Additional file 1: Fig. S2D). Moreover, we observed that meningitic *E. coli* RS218-caused time-dependent downregulation of ZO-1 and Gli2 was totally suppressed by the pre-treatment of EGTA and the PKA inhibitor H89 (Fig. 7C), and the same results were also observed with HlyA and pro-HlyA treatment (Additional file 1: Fig. S2C). Therefore, these findings largely supported that the Ca^2+^ influx and PKA activation induced by α-hemolysin were the reason for the decreased expression of Gli2 and ZO-1 herein. Noticeably, we above showed that meningitic *E. coli* attenuating TGFBRII largely disturbed the TGFβ1-mediated astrocytes-endothelium communication, we additionally found that this TGFBRII reduction could also be mediated by α-hemolysin, by the demonstration that the RS218-WT and RS218-Δ*hlyA*-p*hlyCA* significantly decreased the TGFBRII in hBMECs, while the α-hemolysin deletion mutant RS218-Δ*hlyA* did not (Additional file 1: Fig. S3). Meanwhile, both Ca^2+^ chelator EGTA and PKA inhibitor H89 could not stop this reduction, suggesting that the α-hemolysin-caused TGFBRII reduction was independent of Ca^2+^ influx and PKA activation (Additional file 1: Fig. S3). Anyway, these data revealed that α-hemolysin-triggered Ca^2+^ influx and PKA activation accounted for the decreased expression of Gli2 and ZO-1 in hBMECs.

### Hedgehog signaling agonist SAG protected the BBB integrity from being disrupted by meningitic ***E. coli***

**Fig. 8:**
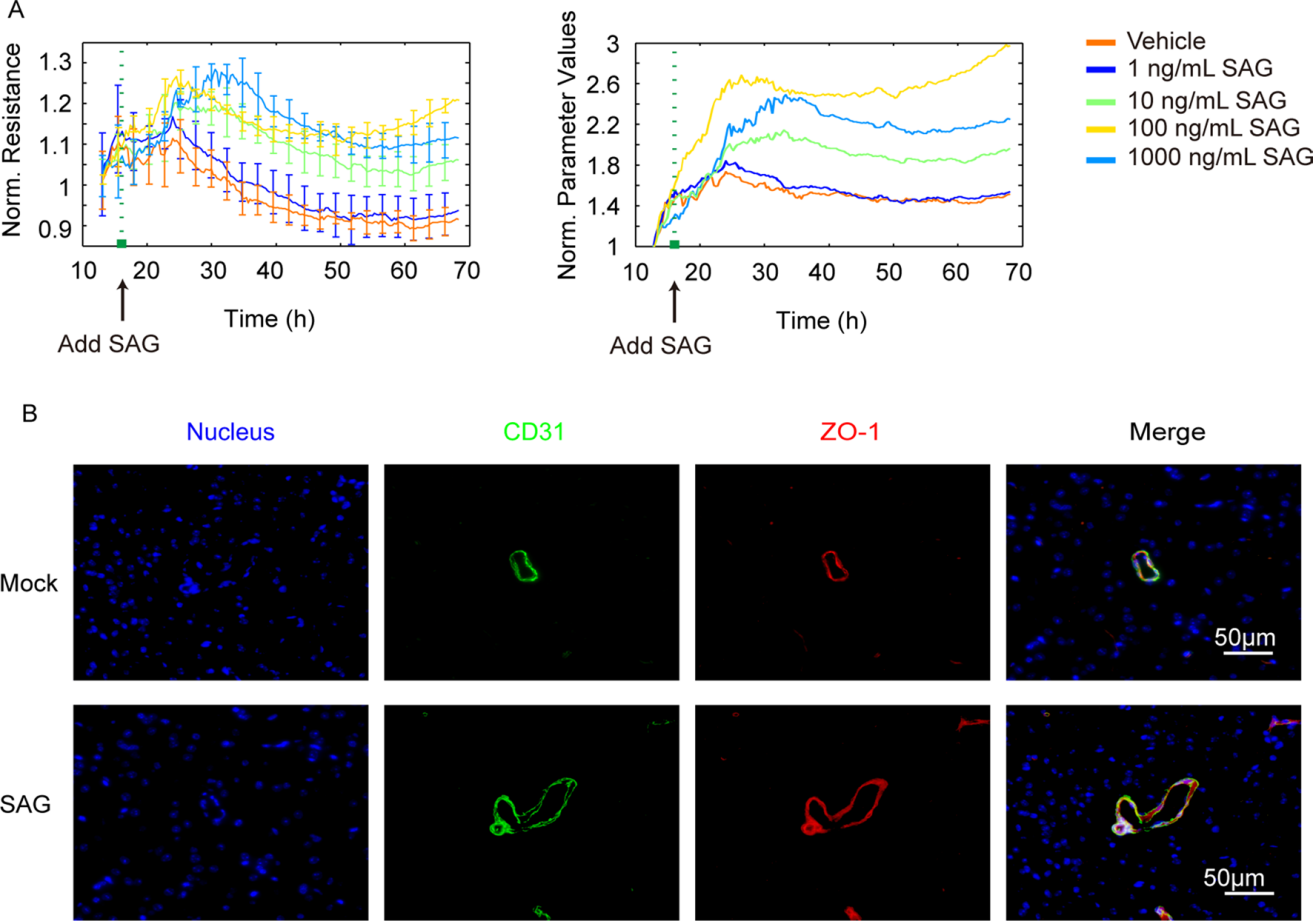
SAG enhanced the barrier function of BMECs. **A** The effect of SAG treatment at different dosages on the barrier resistance *via* ECIS. The left panel indicated the TEER value of hBMECs, and the right panel, representing the factored parameter from TEER values, indicated the barrier function of hBMECs. Each treatment contained 5 replicates, and data were presented as mean ± SEM. **B** The effect of SAG treatment (10 mg/kg) on the ZO-1 expression in mice BMECs. Scale bar indicated 50 μm

**Fig. 9:**
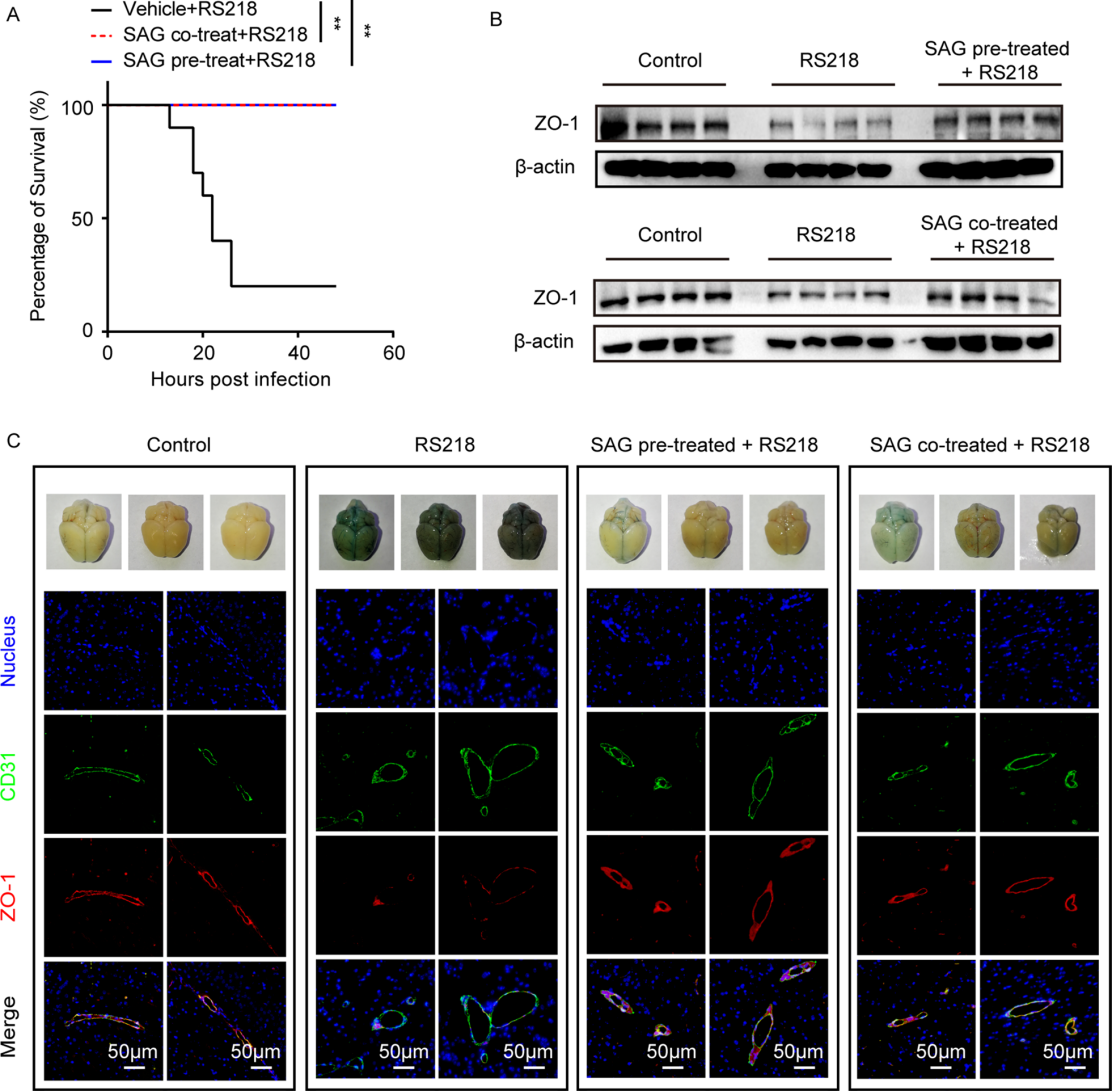
SAG treatment showed good BBB protection in response to RS218 infection. **A** Both pre-treatment (for 12 h) and co-treatment (synchronously) of SAG (10 mg/kg) provided the promising protection in mice against RS218 infection (n = 10). **p < 0.01. **B** The effect of both SAG treatments (10 mg/kg) on the RS218-caused decrease of ZO-1 in mice brains. **C** Evan’s blue and IF assays assessing the SAG-induced protective effects on the BBB permeability as well as the preventive effects on the infection-caused ZO-1 reduction, respectively. Scale bar indicated 50 μm

Since we have evidenced the importance of TGFβ1-triggered hedgehog signaling in astrocytes-endothelium communication and BBB function maintaining [13], we thus hope to see whether activating hedgehog signaling by the known agonist SAG could show some protective effects against bacterial challenge. Here, we firstly tested the possible effects of SAG treatment on the hBMECs barrier function *in vitro*. As the ECIS results shown, SAG effectively increased the barrier resistance of monolayer hBMECs in a dose-dependent manner (Fig. 8A), implying a potential barrier protection role of SAG. *In vivo*, by intravenous injection of SAG, the TJ protein ZO-1 in mice brain presented a high expression around the blood vessels (labeled with CD31) (Fig. 8B). We moreover evaluated the effect of SAG administration in the challenge of RS218 and found that both pre-treatment and co-treatment of SAG with RS218 challenge exhibited promising protective effects in mice (Fig. 9A), and both SAG administration methods significantly attenuated the RS218 infection-induced decrease of ZO-1 expression in mice brains (Fig. 9B). Meanwhile, the brain permeability assays with Evan’s blue showed that both SAG treatments could significantly protect the BBB from being disrupted by the infection, and the IF showed that RS218 infection-caused downregulation of ZO-1 in mice BMECs were significantly prevented by both pre-treatment and co-treatment of SAG (Fig. 9C). These data suggested that activating the hedgehog signaling in BMECs has great potential in the protection of BBB, as well as prevention and control of meningitic *E. coli* infection.

## Discussion

The intercellular communication between astrocytes and endothelium is essential for BBB integrity and CNS homeostasis. Previously, we observed an increased TEER of BMECs *in vitro* when co-cultured with astrocytes U251, and demonstrated that astrocytes-derived TGFβ1 could physiologically help to maintain BBB integrity and function through stabilizing ZO-1 expression in BMECs, in a hedgehog signaling-dependent manner [13]. Since astrocytes-derived TGFβ1 exhibited an endothelial barrier protective effect, while our early studies and data in this work (Fig. 1) supported the disruptive outcomes of the endothelial barrier caused by meningitic *E. coli* infection [21, 29], then there come the questions that whether and how meningitic *E. coli* hijacks this TGFβ1-mediated barrier-maintaining pathway (TGFβ1-TGFBRI/II-Smads-Gli1/2-ZO-1 axis) for its BBB penetration. Here, we demonstrated the TGFβ1 receptor TGFBRII, as well as the hedgehog signaling key transcription factor Gli1/2, were targeted by meningitic *E. coli*. Specifically, on the one hand, the expression of TGFBRII was shown to be significantly decreased in BMECs by the infection of meningitic *E. coli* RS218, during which the transcription factor Sp1 mediated this TGFBRII downregulation. On the other hand, the Gli1/2 in BMECs was also shown to be affected by RS218 challenge, supported by the significantly reduced Gli2 expression and Gli1 nucleus translocation. Such a dual-targeting strategy completely blocked the communication between endothelial cells and astrocytes mediated by TGFβ1, which led to the destruction of BBB caused by meningitic *E. coli*.

We next investigated how RS218 targeted this non-canonical hedgehog activation for the BBB disruption. Since Gli1/2 act as the key transcription factor of hedgehog signaling and Gli2 was observed herein to be significantly decreased in response to RS218, we therefore constructed the Tn5-transposon mutant library in RS218 to screen the key bacterial determinant that targeting Gli2. Fortunately, our screening results specifically pointed to the α-hemolysin operon *hlyCABD*, one of the RTX family members, as the main virulence factor of RS218 to decrease Gli2, thus led to a decrease of ZO-1 expression as well. RTX family were a group of exoproteins secreted from Gram-negative bacteria *via* the type I secretion system (T1SS), with glycine-aspartate (GD)-rich nonapeptide repeats of the consensus sequence G-G-X-G-(N/D)-D-x-(L/I/F)-X near C-terminus [33], and their secretion, maturation, and function were closely related to Ca^2+^ binding. When the toxin was exported outside the T1SS conduit, the high concentration of extracellular Ca^2+^ promoted the correct folding of the C-terminal and prevented its backsliding in the conduit [34]. The binding and perforation of the toxin on the membrane were considered to be receptor-independent. Once perforated on the host cell membrane, the toxin may trigger the influx of Ca^2+^ [35]. As expected, we observed that α-hemolysin HlyA induced the Ca^2+^ influx, leading to the intracellular PKA activation and finally the Gli2 as well as ZO-1 reduction in hBMECs. Noticeably, we also observed a slight Ca^2+^ alteration in hBMECs by the treatment of pro-HlyA, which might due to the weak hemolytic activity of pro-HlyA without the HlyC acylation [36]. For PKA, it was considered a typical inhibitor of Gli2, along with glycogen synthase kinase 3β (GSK-3β) and casein kinase 1 (CK1) [37, 38]. Previous studies in *Drosophila* have shown that PKA could phosphorylate Ci (the alias of Gli in *Drosophila*) at multiple serine/threonine (Ser/Thr) residues of the C-terminal region, and the GSK-3β and CK1 phosphorylation of Ci might be primed by PKA phosphorylation nearby the Ser/Thr residues. The phosphorylated Ci region created the binding site of the so-called SCF (Skp1, Cdc53, and F-box) complex, one of the ubiquitin ligase complexes that normally targets phosphorylated substrates, and finally led to the degradation of Ci [39]. And in mammals, the PKA, GSK-3β, and CK1 were also observed to modulate Gli2 *via* phosphorylation in a similar manner [40]. Therefore, these data together with the Tn5-transposon library screening basically revealed the mechanism of meningitic *E. coli* disturbing the intercellular communication between astrocytes and BMECs. In brief, meningitic *E. coli* exported α-hemolysin HlyA to perforate and trigger the subsequent Ca^2+^ influx and PKA activation, finally led to Gli2 and ZO-1 degradation in hBMECs. Surprisingly, we additionally observed that α-hemolysin HlyA also participated in the RS218-targeted reduction of TGFBRII, but in a Ca^2+^-PKA independent manner. The underlying mechanism of this HlyA-associated TGFBRII reduction was still unclear at this time, but anyway, these findings all supported that α-hemolysin facilitated meningitic *E. coli* subversion of the astrocytes-endothelium communication by attenuating TGFβ1-mediated non-canonical hedgehog signaling.

Both astrocytes-derived TGFβ1 and the hedgehog agonist SAG were previously reported to maintain the vascular and BBB stabilization under stroke or HIV infection [41–43]. As an extension of this study, we also discussed the potential protection of rTGFβ1 and SAG in mice against meningitic *E. coli* infection with two routes of administration, prior to-challenge administration and simultaneous administration. For rTGFβ1 (Fig. 1C), pre-administration of rTGFβ1 better protected the challenged mice, while in contrast, treatment with rTGFβ1 while challenged did not reverse the death of challenged mice. This outcome may be attributed to the significant downregulation of the TGFβ1 receptor TGFBRII induced by meningitic *E. coli*, and the simultaneous TGFβ1 treatment could not exert the timely and effective barrier protection effect due to the lack of its receptor. In contrast, for hedgehog agonist SAG (Fig. 9), both pre-treatment and co-treatment of SAG in mice, all exhibited the protective effects against RS218 challenge, which supported a promising potential of SAG in control of the infection, especially when applied simultaneously. This remarkable effect was further supported by the significant recovery of the infection-caused ZO-1 decrease and the BBB disruption in mice brains with both routes of SAG treatment. Besides, the previous work has also demonstrated that activating hedgehog signaling in BBB could reduce Th1 and Th17 cells’ adherence to BMECs and suppress neuroinflammatory responses, thus helping maintain CNS immune quiescence [44]. These reports, together with our observations herein, largely suggested that activating hedgehog signaling by SAG in BMECs would be beneficial for the BBB protection, as well as for the prevention and control of bacterial-induced CNS dysfunction.

**Fig. 10:**
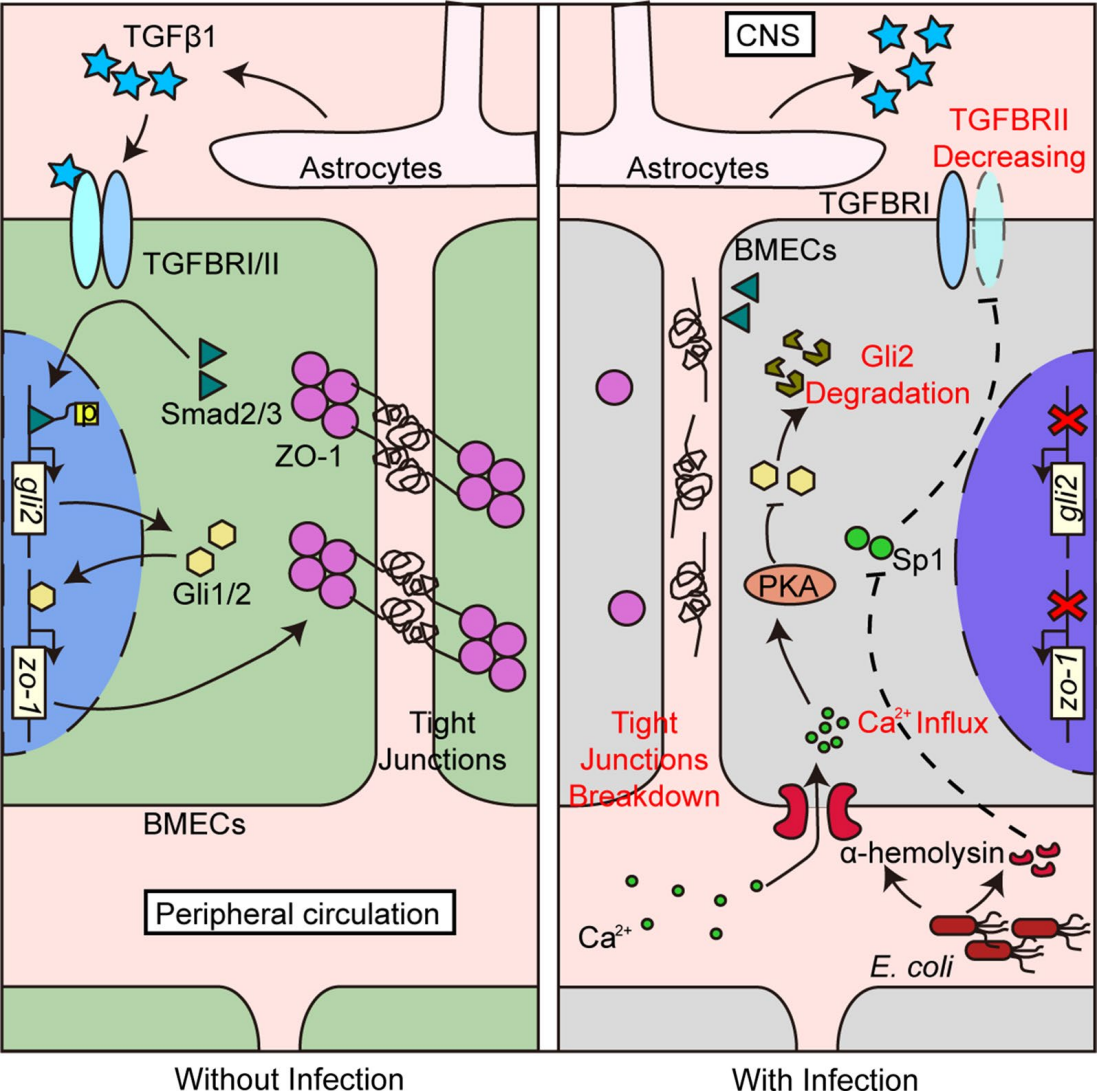
Schematic presentation of the TGFβ1-mediated intercellular communication for maintaining the normal BBB function as well as the disturbance by meningitic *E. coli* for the BBB disruption. Without infection, astrocytes-derived TGFβ1 physiologically triggered the TGFβ1-TGFBRII-Smad2/3-Gli1/2-ZO-1 axis in BMECs to maintain the normal BBB function (left panel). Upon meningitic *E. coli* infection, bacterial virulence determinant α-hemolysin effectively helped to disturb this functional cross-talking between astrocytes and BMECs by attenuating both TGFβ1 receptor TGFBRII as well as the hedgehog signaling transcription factors Gli1/2 in BMECs, thus led to the BBB dysfunction

In summary, together with our previous finding, we demonstrated a TGFβ1-mediated cell-to-cell communication for maintaining the normal BBB function, as well as its disturbance by meningitic *E. coli* for the BBB disruption. As presented in Fig. 10, the astrocytes-derived TGFβ1 triggered the TGFβ1-TGFBRII-Smad2/3-Gli1/2-ZO-1 axis in BMECs which accounted for the physiological BBB function maintaining [13], while meningitic *E. coli* craftily disturbed this intercellular cross-talking by attenuating both TGFβ1 receptor TGFBRII and the key transcription factors of hedgehog signaling Gli1/2 in BMECs, which largely depended on the action of α-hemolysin. Strikingly, the hedgehog signaling agonist SAG presented powerful protection of the BBB integrity and the mice survival from meningitic *E. coli* challenge. Together, these findings reveal a novel pathogenic mechanism in meningitic *E. coli*-caused BBB disruption from the perspective of intercellular communication between astrocytes and vascular endothelium within BBB, and highlight the critical roles of hedgehog signaling in preventing the CNS dysfunction caused by bacterial infection.

## Supplementary Information

**Additional file 1.** Additional figures and tables.

## Data Availability

All data generated or analyzed during this study are included in this published article and its additional information files.

## Abbreviations

CNS: Central nervous system
*E. coli*: *Escherichia coli*
BBB: Blood–brain barrier
BMECs: Brain microvascular endothelial cells
hBMECs: Human brain microvascular endothelial cells
TEER: Trans-endothelial electrical resistance
TGFβ1: Transforming growth factor-β1
Hly: Hemolysin
RTX: Repeats-in-toxin
UPEC: Uropathogenic *E. coli*
DAPI: 4′,6-diamidino-2-phenylindole
ECIS: Electric cell-substrate impedance sensing
CDS: Coding sequence
ChIP: Chromatin immunoprecipitation
TAIL-PCR: Thermal asymmetric interlaced PCR
PKA: Protein kinase A
EGTA: Ethyleneglycolbis(2-aminoethylether)-N,N,N′,N′-tetraaceticacid
