# Supplementary material for: Meningitic Escherichia coli α-hemolysin aggravates blood–brain barrier disruption via targeting TGFβ1-triggered hedgehog signaling
Source: Mol Brain. 2021 Jul 19;14:116. doi: 10.1186/s13041-021-00826-2 (PMC8287823; doi:10.1186/s13041-021-00826-2)
Supplement: Supplementary file 1 — Additional file 1. Additional figures and tables. [file 13041_2021_826_MOESM1_ESM.docx]

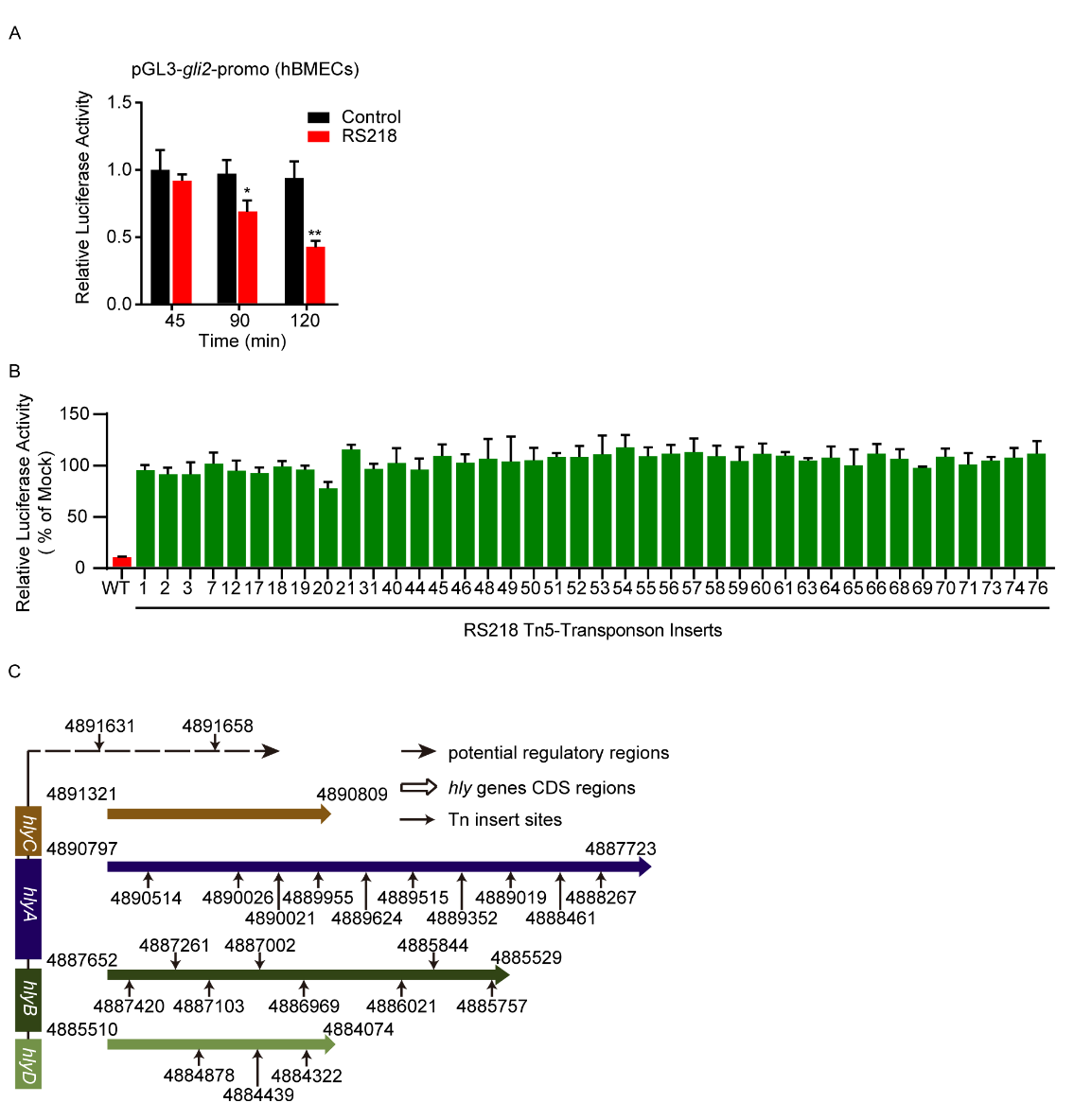


**Fig. S1** High-throughput screening of Tn5-transposon mutation library identified that α-hemolysin participated in the regulation of *gli2* promotor activity. **(A)** Firefly-luciferase reporter assay testing the effect of RS218 infection on the *gli2* promoter activity in hBMECs. Results were obtained from three independent assays and presented as mean ± SEM. ***p*<0.01. **p*<0.05. **(B)** Firefly luciferase activity screening showing the Tn5-transposon inserts unable to reduce the *gli2* promotor activity. RS218-WT strain was tested as positive control. Data were presented as mean ± SEM from three independent replicates. **(C)** Schematic of the genome locations showed that the positive Tn5-transposon inserts mainly located in the *hlyCABD* operon.


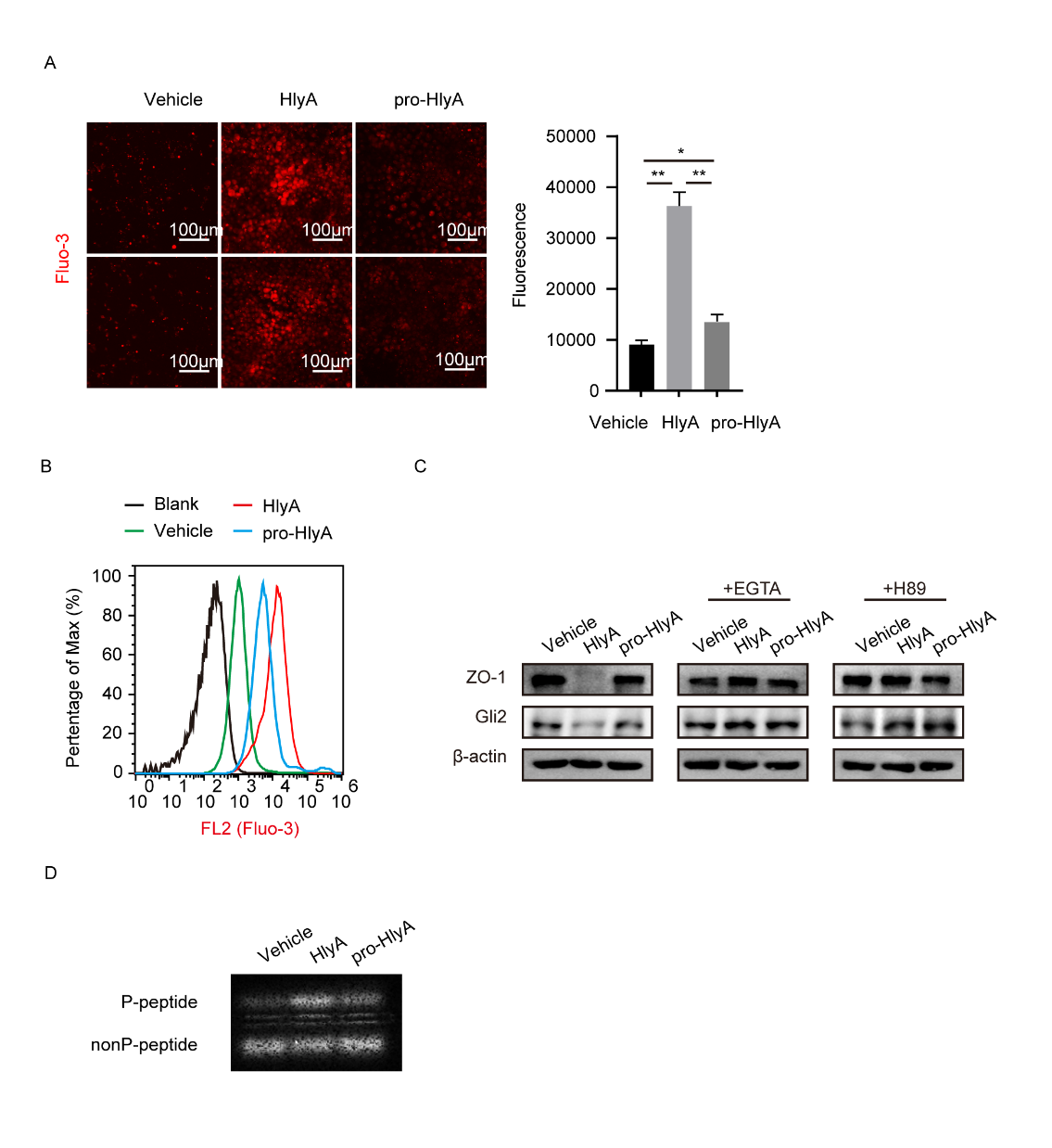


**Fig. S2** The activity of mature HlyA was indispensable for Ca^2+^ influx and PKA activation as well as ZO-1 and Gli2 reduction. **(A-B)** The Ca^2+^ influx of hBMECs in response to HlyA (1μg/mL) or pro-HlyA (1μg/mL) for 2 h determined by the Fluo-3-AM probed through fluorescence microscopy **(A)** as well as flow cytometry **(B)**. The fluorescence intensity was calculated from 3 independent measurements and presented as mean ± SEM. * *p*<0.05, ** *p*<0.01 compared with vehicle. Scale bar indicated 100 μm. Cells in Blank group were not treated. Cells in Vehicle group, HlyA group, and pro-HlyA group were loaded with Fluo-3-AM and treated as indicated. **(C)** The expression of ZO-1 and Gli2 in hBMECs in response to HlyA (1μg/mL) or pro-HlyA (1μg/mL) for 2 h, as well as those with pretreatment of Ca^2+^ chelator EGTA (5mM) or PKA activity inhibitor H89 (20μM). **(D)** PKA activity in hBMECs treated with HlyA (1μg/mL) or pro-HlyA (1μg/mL) for 2 h.


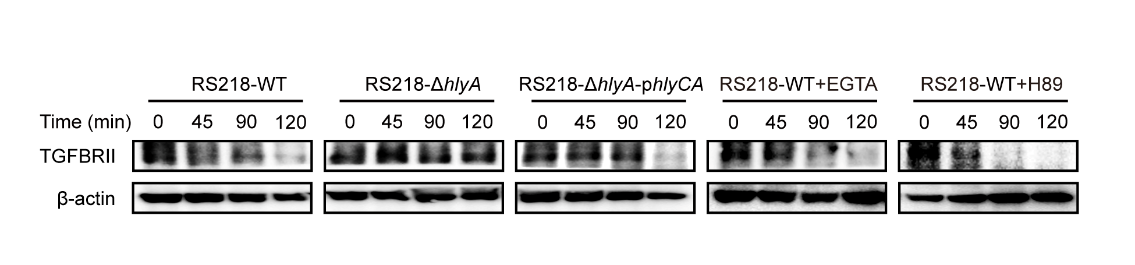


**Fig. S3** The HlyA-triggered Ca^2+^ influx and PKA activation were involved in the infection-caused TGFBRII reduction. The TGFBRII expression in hBMECs was time-dependently decreased in response to the infection by RS218-WT and RS218-*ΔhlyA*-p*hlyCA* strains, but not the infection by RS218-*ΔhlyA*. And both EGTA or H89 treatment could not prevent the infection-caused reduction of TGFBRII.

**Supplemental Tables**

**Table S1.** The targets of the positive Tn5-Transposon insertions in RS218 genome.

| *Genes* | Insertions frequency |
| --- | --- |
| *hlyA* | 10 |
| *hlyB* | 8 |
| *hlyD* | 3 |
| regulation regions upstream *hlyC* | 2 |
| *guaA* (pseudo) | 1 |
| *tpi* | 1 |
| *purH* | 1 |
| *integrase* | 1 |
| unknown sequence | 1 |
| hypothetic protein | 1 |
| In Total | 29 |

**Table S2.** Primers used for the qPCR assays.

| Primer name | Sequence (5’ to 3’) |
| --- | --- |
| *tgfbr2*-F | GTGTGCCAACAACATCAA |
| *tgfbr2*-R | GTCCTTCTCTGTCTTCCAA |
| *sp1*-F | CTACTACCACCAGCAACA |
| *sp1*-R | AATGAGCCTCCAGATGTC |
| *gli1*-F | GCTAGAGTCCAGAGGTTC |
| *gli1*-R | GTGGTGAGTAGACAGAGG |
| *gli2*-F | AGCAGCAGCAACTGTCTGAGTGA |
| *gli2*-R | GACCTTGCTGCGCTTGTGAA |
| *gapdh*-F | CAACAGCCTCAAGATCATCAG |
| *gapdh*-R | GAGTCCTTCCACGATACCA |

**Table S3.** Primers for CDSs cloning and promotor region amplification in the dual-luciferase reporter assays.

| Primer name | Sequence (5’ to 3’) |
| --- | --- |
| Sp1-CDS -F | CCCAAGCTTGTCCCTCAGCTGCCACCATG |
| Sp1-CDS -R | CCGGAATTCGCCCCGGGTGCCTGATCTCA |
| *tgfbr2*-promo-F | CGAGCTCTGGTGGCAGAAGAGGGAATAG |
| *tgfbr2*-promo-R | CCCAAGCTTACTCACTCAACTTCAACTCAGC |
| *tgfbr2*-promo truncation1-F | CGAGCTCGCCTCTGCAATCTTCCTCTCA |
| *tgfbr2*-promo truncation1-R | CCCAAGCTTACTCACTCAACTTCAACTCAGC |
| *tgfbr2*-promo truncation2-F | CGAGCTCATTCAGGTGACTCCCAACCC |
| *tgfbr2*-promo truncation2-R | CCCAAGCTTACTCACTCAACTTCAACTCAGC |
| *tgfbr2*-promo truncation3-F | CGAGCTCGCATATAGACACAACTGAAGCA |
| *tgfbr2*-promo truncation3-R | CCCAAGCTTACTCACTCAACTTCAACTCAGC |
| *tgfbr2*-promo truncation4-F | CGAGCTCCATGATTGGCAGCTACGAGAG |
| *tgfbr2*-promo truncation4-R | CCCAAGCTTACTCACTCAACTTCAACTCAGC |
| *tgfbr2*-promo mutation1-F | TGGGAGTTGGGTTTGATCCGTTCATATTTACCTTTATTCCCT |
| *tgfbr2*-promo mutation1-R | ACCCTCAACCCAAACTAGGCAAGTATAAATGGAAATAAGGGA |
| *tgfbr2*-promo mutation2-F | GAACTCCTGAGTGGTGAATAAGTATGGTGAGGGGCAGCTGA |
| *tgfbr2*-promo mutation2-R | CTTGAGGACTCACCACTTATTCATACCACTCCCCGTCGACT |
| *tgfbr2*-promo mutation3-F | AGAGGGAGAAGGCTCTAGTGTACAATGAGGTCCTGCCCAGC |
| *tgfbr2*-promo mutation3-R | TCTCCCTCTTCCGAGATCACATGTTACTCCAGGACGGGTCG |

**Table S4.** Primers used in ChIP-PCR/qPCR assays.

| Primer name | Sequence (5’ to 3’) |
| --- | --- |
| ChIP-SP1×*tgfbr2*-F | GGCTGGTCTAGGAAACAT |
| ChIP-SP1×*tgfbr2*-R | GACTCACTCAACTTCAACTC |

**Table S5.** Primers used in the TAIL-PCR.

| Primer name | Sequence (5’ to 3’) |
| --- | --- |
| Tn-F1 | GGATTGGCGTAAGCGTGCGGGATAT |
| Tn-F2 | TCTGAAATCCATCCCTGTCGGTGTTGCTTAT |
| Tn-F3 | GCGAGATCACCAAGGTAGTCGGCAAATAATG |
| Tn-F4 | TTGCTCACAGCCAAACTATCAGGTCAAGTC |
| Tn-R | GATCCGACGTGTTGCACACTAGTAC |
| ADR-1 | GATCCGACGTGTTGCACACTAGTACNNNNNNCTTAT |
| ADR-2 | GATCCGACGTGTTGCACACTAGTACNNNNNNCCGAA |
| ADR-3 | GATCCGACGTGTTGCACACTAGTACNNNNNNGGAA |
| ADR-4 | GATCCGACGTGTTGCACACTAGTACNNNNNNTGGTT |
| ADR-5 | GATCCGACGTGTTGCACACTAGTACNNNNNNCCAA |
| ADR-6 | GATCCGACGTGTTGCACACTAGTACNNNNNNCGGT |

**Table S6.** Primers used for the *hly* deletion.

| Primer name | Sequence (5’ to 3’) |
| --- | --- |
| pTargetF-*hlyC*-F | ACTATTCCGAGCCATCAGGGGTTTTAGAGCTAGAAATAGC |
| pTargetF-*hlyC*-R | CCCTGATGGCTCGGAATAGTACTAGTATTATACCTAGGAC |
| *hlyC*-HAL-F | CCGATATTATCAAGGTTAGGT |
| *hlyC*-HAL-R | ATGAACAATCCATTAGAGGTTCATTAACAGGTTAAGAGGTAA |
| *hlyC*-HAR-F | ACCTCTAATGGATTGTTCATAT |
| *hlyC*-HAR-R | CGTCTCTGGTTGAATTGA |
| pTargetF-*hlyA*-F | GCAATGGACAGGAATGAGAGGTTTTAGAGCTAGAAATAGC |
| pTargetF-*hlyA*-R | CTCTCATTCCTGTCCATTGCACTAGTATTATACCTAGGAC |
| *hlyA*-HAL-F | CACCACCACAACAGATAA |
| *hlyA*-HAL-R | AAATGCCAACAATAACCACTTTGACAGCATCAGCATAA |
| *hlyA*-HAR-F | AGTGGTTATTGTTGGCATT |
| *hlyA*-HAR-R | TTATGTTAATGTGCGTTATGAA |
| pTargetF-*hlyB*-F | GAGAGTTAAGCACATCACCAGTTTTAGAGCTAGAAATAGC |
| pTargetF-*hlyB*-R | TGGTGATGTGCTTAACTCTCACTAGTATTATACCTAGGAC |
| *hlyB*-HAL-F | AATAAGGCTCATCAGGAAG |
| *hlyB*-HAL-R | TGGAGTCATAATGGATTCTTTAACAGAAAGAACAGAAGAATATG |
| *hlyB*-HAR-F | AAGAATCCATTATGACTCCAA |
| *hlyB*-HAR-R | GGGAAAGACGATAAACTCA |
| pTargetF-*hlyD*-F | AGGTGGGGTTGTTACAACAGGTTTTAGAGCTAGAAATAGC |
| pTargetF-*hlyD*-R | CTGTTGTAACAACCCCACCTACTAGTATTATACCTAGGAC |
| *hlyD*-HAL-F | CCATACAGAACAGCATTATATC |
| *hlyD*-HAL-R | AAAGAACAGAAGAATATGAAGAGCGTTAAGTCTCAGAG |
| *hlyD*-HAR-F | TTCATATTCTTCTGTTCTTTCTG |
| *hlyD*-HAR-R | CGATCCTAACTGGTTACG |
| pMD19-*hlyCA* | ATGAATATGAACAATCCATTAGAGGTTC |
| pMD19-*hlyCA* | TAAGAGTGCTGATGCTGTCAAAGTTATTGA |

**Table S7.** Primers used for the *hlyA* and *hlyCA* Cloning.

| Primer name | Sequence (5’ to 3’) |
| --- | --- |
| pET28a-*hlyA*-F | CCCGAGCTCATGCCAACAATAACCACTGCAC |
| pET28a-*hlyA*-R | CCGCTCGAGTGCTGATGCTGTCAAAGTTATTGA |
| pET28a-*hlyCA*-F | CCCGAGCTCATGAATATGAACAATCCATTAGAGGTTC |
| pET28a-*hlyCA*-R | CCGCTCGAGTGCTGATGCTGTCAAAGTTATTGA |
